# Unraveling Colorectal Cancer and Pan-cancer Immune Heterogeneity and Synthetic Therapy Response Using Cuproptosis and Hypoxia Regulators by Multi-omic Analysis and Experimental Validation

**DOI:** 10.7150/ijbs.84781

**Published:** 2023-07-09

**Authors:** Pei-Cheng Jiang, Jin Fan, Chun-Dong Zhang, Ming-Hua Bai, Quan-Quan Sun, Qian-Ping Chen, Wei Mao, Bu-Fu Tang, Hui-Yin Lan, Yang-Yang Zhou, Ji Zhu

**Affiliations:** 1 Department of Radiation Oncology, Zhejiang Cancer Hospital, Hangzhou, China.; 2 Hangzhou Institute of Medicine (HIM), Chinese Academy of Sciences, Hangzhou, China.; 3 Department of Radiation Oncology, Fudan University Shanghai Cancer Center, Shanghai, China.; 4 Department of Gastrointestinal Surgery, The Fourth Affiliated Hospital of China Medical University, Shenyang, China.; 5 Department of Gastrointestinal Surgery, Graduate School of Medicine, The University of Tokyo, Tokyo, Japan.; 6 Zhejiang Key Laboratory of Radiation Oncology, Hangzhou, China.; 7 Department of Radiation Oncology, Zhongshan Hospital Affiliated to Fudan University, Shanghai, China.; 8 Department of Oncology, Shanghai Sixth People's Hospital affiliated to Shanghai Jiao Tong University School of Medicine, Shanghai, China.

**Keywords:** cuproptosis, hypoxia, clustering, colorectal cancer, precision treatment

## Abstract

Cuproptosis, a new type of programmed cell death (PCD), is closely related to cellular tricarboxylic acid cycle and cellular respiration, while hypoxia can modulate PCD. However, their combined contribution to tumor subtyping remains unexplored. Here, we applied a multi-omics approach to classify TCGA_COADREAD based on cuproptosis and hypoxia. The classification was validated in three colorectal cancer (CRC) cohorts and extended to a pan-cancer analysis. The results demonstrated that pan-cancers, including CRC, could be divided into three distinct subgroups (cuproptosis-hypoxia subtypes, CHSs): CHS1 had active metabolism and poor immune infiltration but low fibrosis; CHS3 had contrasting characteristics with CHS1; CHS2 was intermediate. CHS1 may respond well to cuproptosis inducers, and CHS3 may benefit from a combination of immunotherapy and anti-fibrosis/anti-hypoxia therapies. In CRC, the CHSs also showed a significant difference in prognosis and sensitivity to classic drugs. Organoid-based drug sensitivity assays validated the results of transcriptomics. Cell-based assays indicated that masitinib and simvastatin had specific effects on CHS1 and CHS3, respectively. A user-friendly website based on the classifier was developed (https://fan-app.shinyapps.io/chs_classifier/) for accessibility. Overall, the classifier based on cuproptosis and hypoxia was applicable to most pan-cancers and could aid in personalized cancer therapy.

## Introduction

As an indispensable trace element, copper (Cu) balance is essential for organisms since abnormal Cu metabolism affects cellular respiration, proliferation, and migration 1, 2 and is linked to multiple tumors 3. The influence of Cu on tumors is generally believed to occur through the regulation of intracellular redox reactions 4. Until 2022, aberrant intracellular Cu have been found to mediate programmed cell death (PCD), but the underlying mechanism is distinct from previously known forms of PCD. Excessive Cu binds directly to lipid-acylated components of the tricarboxylic acid (TCA) cycle, causing the accumulation of lipid-acylated proteins and loss of iron-sulfur cluster proteins, resulting in cell death. Additionally, it has been observed that cells reliant on mitochondrial respiration are significantly more sensitive to Cu ionophores than those reliant on glycolysis, revealing the importance of cellular respiration in cuproptosis 5.

Cellular respiration is dependent on oxygen levels in intracellular environment. The deleterious biological effects of hypoxia in tumors have been widely recognized 6. Hypoxia affects tumor behavior through modulating of the physicochemical environment and intracellular signaling transduction pathways 7. Moreover, hypoxia has been found to regulate multiple types of PCD 8. Tsvetkov et al. also observed that while stabilization of the HIF pathway did not alleviate cuproptosis, improving the hypoxic environment did 5. Given that cuproptosis is a novel form of PCD, the interplay between it and hypoxia is a topic that merits further exploration.

Cu overload is associated with the progression and prognosis of colorectal cancer (CRC) 9. But the underlying mechanisms remain poorly understood. As for hypoxia, it has impact on various aspects of CRC, including altering the cellular metabolism, tumor microenvironment (TME), and radiotherapy sensitivity 10, 11.

Heterogeneity is common in cancers that poses a significant obstacle to precision therapy 12,13. Therefore, a classification system capable of identifying patient subgroups with distinct features would facilitate personalized treatments. Herein, we hypothesize that multi-omics analysis of patients with varying hypoxic and cuproptotic statuses may help elucidate the disparities and links among individuals, offering novel insights into tumor classification and treatment.

## Materials and methods

**Figure 1:**
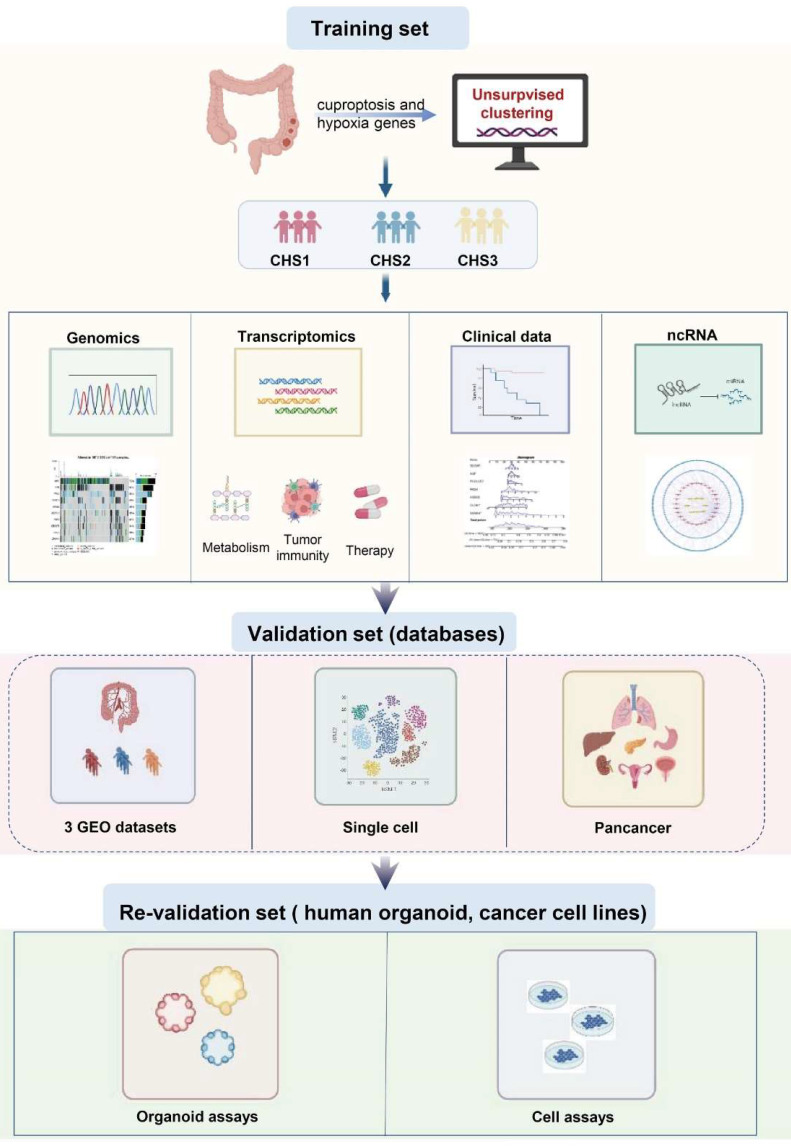
The flowchart of the study.

The flow diagram of the study is shown in Figure 1.

### Patient cohorts and cell lines

Transcriptomic, non-coding RNA and methylation data of TCGA_COADREAD were downloaded from the USCS Xena portal. Transcriptomic data of pan-cancer were obtained from pan-cancer subsets on XENA, normalized by log2 (X+1). After removing non-tumor samples, 9,185 samples were reserved for analysis.

A total of 1,281 CRC patients from three Gene Expression Omnibus (GEO) databases were recruited (accession #GSE39582, #GSE196576, and #GSE28702). Also, the single-cell RNA (scRNA) dataset GSE144735, including 8,254 cells, was downloaded.

Four CRC cell lines, HCT116, LS180, HT29, and SW620, were purchased from the Institute of Biochemistry and Cell Biology, Chinese Academy of Sciences, Shanghai, China. All cells were cultured in high-glucose Dulbecco's modified eagle's medium (DMEM) (KeyGEN, China) with 10% fetal bovine serum (FBS) (Hyclone, USA) and 1% penicillin/streptomycin solution (GIBCO, USA) at 37 °C in a 5% CO_2_ incubator. Masoprocol, forskolin, kenpaullone, pazopanib, masitinib, simvastatin, vorinostat, MK-2206, and MK-0752 were purchased from MedChemExpress (USA). Idarubicin was purchased from TargetMol (China).

Twenty-eight CRC samples were collected from Zhejiang Cancer Hospital for organoid culture. This study was approved by the Institutional Review Boards of Zhejiang Cancer Hospital (IRB-2021-291) and performed in accordance with the recognized ethical guidelines of the Declaration of Helsinki. All participants signed informed consent forms.

The profiles of the datasets in this study are listed in Table 1.

### Consensus molecular clustering

The key genes for cuproptosis were obtained from the literature 5, and the key genes for hypoxia were obtained from the hallmark of the Molecular Signatures Database (MSigDB), excluding genes directly associated with the HIF pathway. The key genes are listed in Table S1. R package “ConsensusClusterPlus” 14 was applied to execute consensus clustering of TCGA_COADREAD. Calculating the Euclidean distances with k-means, we chose three as the optimal cluster. Using TCGA_COADREAD as the training set, we applied the K-nearest neighbor algorithm (KNN) 15 to classify the samples in the validation and re-validation sets.

### Functional enrichment analysis

We applied the "limma" package for the differential analysis. Differential expression genes (DEGs) was determined by identifying genes with fold change of ≥1 and adjusted *P* value (FDR) of ≤0.05. Gene ontology (GO), gene set enrichment analysis (GSEA), and gene set variation analysis (GSVA) were performed with the R package “clusterProfiler” 16 based on gene sets of MsigDB and the Kyoto Encyclopedia of Genes and Genomes (KEGG).

### Immune-related features

We applied multiple algorithms to estimate the relative enrichment of immune cells, including TIMER 2.0 17, EPIC 18, and the microenvironment cell-populations counter (MCP-counter) 19. The Tracking Tumor Immunophenotypes (TIP) was applied to assess the immune cycle 20.

The “ESTIMATE” 21 package was used to calculate the ESTIMATE, stromal, and immune scores. Functional gene expression signatures (Fges) were applied for an overview of the profile of immune characteristics 22.

### Methylation and non-coding RNA (ncRNA) analysis

The collated methylation data were analyzed using the “CHAMP” package 23.

Integrating the interactive information of ncRNA and mRNA in Starbase 24, mirDIP 25 and Targetscan 26 datasets, we obtained hub lncRNAs and corresponding mRNAs. Then, the ceNetwork was constructed with Cytoscope.

### The construction of a cuproptosis-hypoxia subtype (CHS) website

For researches' accessibility, we constructed a website dedicated to CHS analysis using the standard Model-View-Controller pattern with the "Shiny" package.

### Colorectal cancer organoids (CRCOs) assay

Resected colorectal cancer tumor tissues were placed in 4 °C phosphate buffered salin with normocin (Invitrogen, China) and gentamicin/amphoteritin B (GIBCO, USA) for cell isolation. The tissues were cut into tiny fragments after removing blood and necrotic fractions. The tissue fragments were dissociated with digestion buffer (7 mL DMEM) (GIBCO,USA), 500 U/mL collagenase IV (Sigma, USA), 1.5 mg/mL collagenase II (Solarbio, China), 20 mg/mL hyaluronidase (Solarbio, China), 0.1 mg/mL dispase type II (Sigma, USA), 10 uM RHOK inhibitor ly27632 (Sigma, USA), and 1% FBS (Hyclone, USA) on a shaker at 37 °C for 30-60min. After lysing red cells, dissociated single cells were resuspended with Matrigel gel, and were pipetted into pre-warmed 24-well plates. CRCO culture medium (1 mL/well) 27 was added after gel solidification. Next, the plates were incubated in a 37 °C and 5% CO_2_ culture incubator. CRCO culture medium was refreshed every 3 days. To characterize the organoids and validate their fidelity to the original tumor, we performed immunofluorescence staining for colorectal cancer organoid markers, including Ki-67, CDX2, β-catenin, CK20, and CK-pan.

To test the IC50 values of the drugs, 100 organoids with a particle size of 70 um to 100 um were selected and laid in 96-well plate per well. Incubating with CRCO culture medium for 24 h, we replaced the primary culture medium with a new organoids culture medium containing different concentrations of 5-Fu, SN38 or oxaliplatin (Selleck, USA). After 5 days, the organoids viability was evaluated with Cell-Titer-Glo 3D Cell viability assay (Promega, USA) according to the manufacturer's instruction.

### Drug susceptibility analysis

We collected the target genes of CRC drugs from Drugbank 28 and analyzed their expressions. Moreover, we submitted DEGs to the CMap database to predict potential drugs. Next, we determined the connection between the drugs and the key genes using the Cancer Cell Line Encyclopedia database.

### Cell proliferation and migration assay

The cell counting kit-8 assay (BEIERBO, China) was used to detect cell proliferation according to the manufacturer's protocol.

We used 24 Transwell chambers (Corning, USA) for the migration assay. The detected cells were suspended in a serum-free medium, and were inoculated in the upper chambers. The lower chambers contained DMEM with 10% FBS. Incubating for 24 h, we fixed the cells on the bottom side of the membrane with 4% methanol for 15 min after wiping the top side. Then, the fixed cells were stained with 0.1% crystal violet for 15 min at room temperature, photographed by light microscope (Leica, Germany) and manually counted.

### Quantitative Real-time PCR (qPCR)

Total RNA was extracted from the cells using a cellular RNA extraction kit (Vazyme, China) following the manufacturer's instructions. The extracted RNA was then reverse transcribed into complementary DNA (cDNA). For the qPCR reactions, the cDNA, SYBR Green master mix (Vazyme, China), double-distilled water (ddH2O), and gene-specific primers (refer to Table S2) were mixed to the reaction mixture. The reaction mixture was subjected to denaturation, annealing, and extension to complete the amplification process (applied biosystems, USA).

### Western blot

The cells were first placed on ice and lysed to extract cellular proteins. The cell lysate was then subjected to SDS-PAGE (sodium dodecyl sulfate-polyacrylamide gel electrophoresis) to separate the proteins based on their molecular weight. Then, the proteins were transferred onto a PVDF (polyvinylidene fluoride) membrane. To prevent non-specific binding, the PVDF membrane was blocked with non-fat milk. After blocking, the membrane was incubated with specific primary antibodies against the target proteins (abcam, USA). Subsequently, the membrane was incubated with secondary antibodies.

Finally, the chemiluminescent signals were captured by imaging system (Azure Biosystems, California).

### Statistical analysis

All statistical analyses were performed using R software (version 4.1.2). The Kaplan-Meier survival curve was plotted using the 'Survminer' package. Univariate Cox and LASSO Cox regression analyses were applied to establish the prognostic model. The R package “survivalROC” was used for the time-dependent receiver operating characteristic (ROC) curve. The “rms” package was employed to construct nomogram and calibration curves. The linear relationship was measured using Pearson's correlation analysis. The Wilcoxon test was used to compare the differences between the two groups. The analysis of variance test was performed for multiple groups. The survival times were compared using the log-rank test. All *p*-values were two-sided, and values < 0.05 were considered statistically significant.

## Results

### The establishment of cuproptosis-hypoxia subtypes

Using unsupervised clustering, TCGA_COADREAD patients were classified into three clusters, namely CHS1, CHS2, and CHS3, based on the key genes associated with cuproptosis and hypoxia (Figure S1A). CHS1 had the highest scores for cuproptosis and the lowest scores for hypoxia, while CHS3 had the opposite (Figure S1B). For the validation datasets, the score characteristics were consistent with those of the training set (Figure S1F). CHS1 had an earlier stage and fewer metastases, whereas CHS3 had an inverse trend (Figure S1C). However, there were no significant differences among the three subtypes regarding age and sex (Figure S1D-E).

### Mutation characteristics

**Figure 2:**
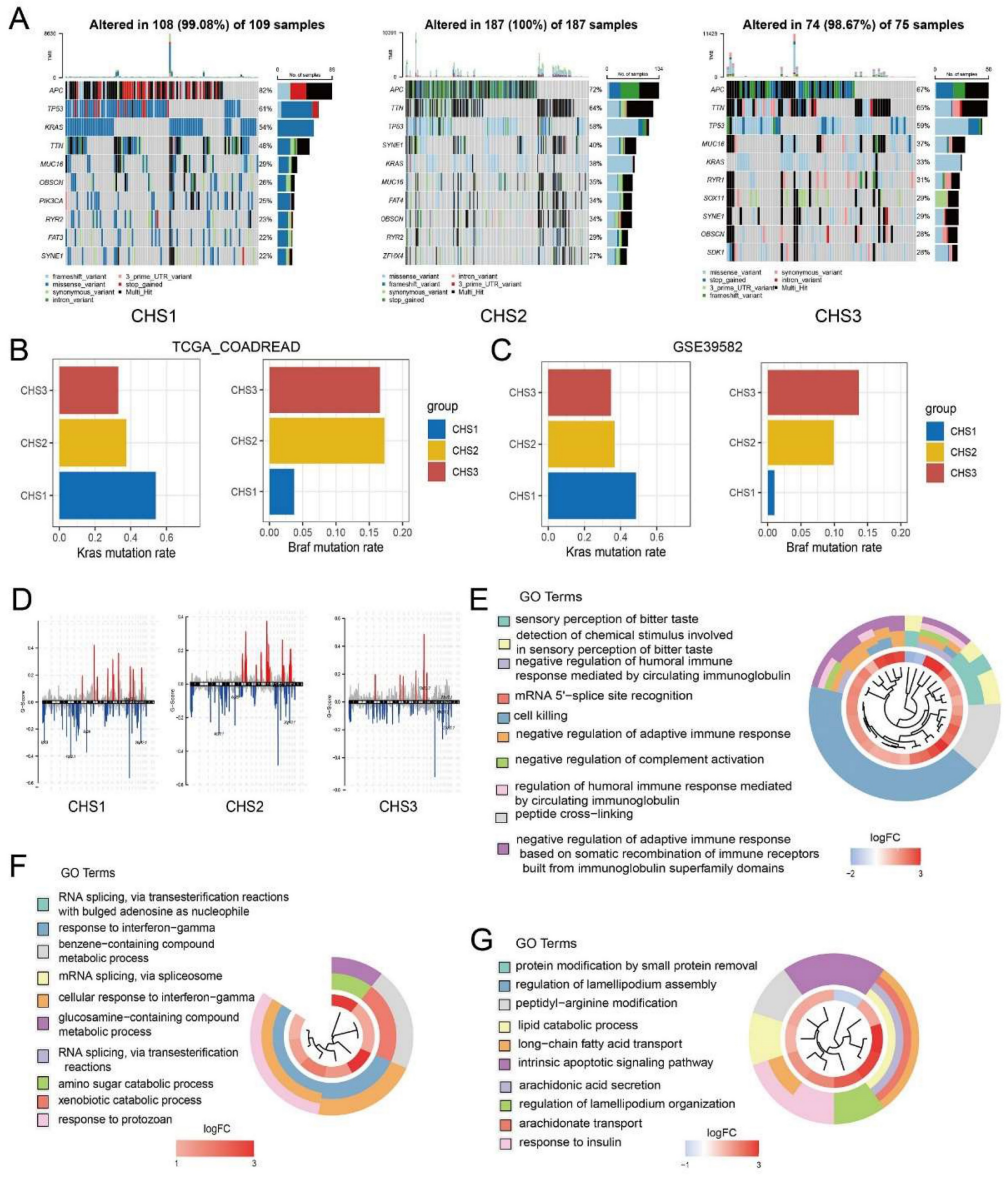
** The genomic characteristics in CHSs.** A. The characteristics of SNP in 3 CHSs. B,C. Kras and Braf mutation rate of CHSs in training set and GSE39582, respectively. D. The characteristics of CNV in CHS1, CHS2, CHS3. E-G. The result of GO analysis to loci with mutation in CHS1, CHS2, CHS3.

Single nucleotide polymorphism (SNP) characteristics presented disparities in the top 10 genes among the CHSs (Figure 2A). The mutation rates varied significantly, even among the genes that were shared among them. For instance, Kras, a recognized oncogene, was found to be mutated in 54% of CHS1 and only 3% of CHS3. Kras mutations in CRC cells altered the demand for Cu bioavailability by varying the intracellular Cu pool 29. Considering the difference in cuproptosis among the CHSs, Kras status may be associated with cuproptosis in CRC. As an unfavorable prognostic factor in CRC, the Braf mutation rate was 3.7% and 17.3% in CHS1 and CHS3, respectively, indicating poor prognosis in CHS3 (Figure 2B). The mutation tendencies of Kras and Braf among CHSs were verified in the validation set (Figure 2C). The proportion of SNP types also differed among the subtypes (Figure S1G).

Copy number variation (CNV) analysis showed that abnormal amplification and deletion varied among the CHSs (Figure 2D). The loci with mutation rate results q < 0.05 were retained for GO enrichment analysis. Loci in CHS1 were enriched for circulating immunoglobulins, adaptive immune responses, and so on. The results of CHS2 included mRNA splicing and cellular responses to INF_a, whereas the results of CHS3 were lipid metabolism, and endogenous apoptosis, among others (Figure 2E-G).

### Profile of hallmarks

**Figure 3:**
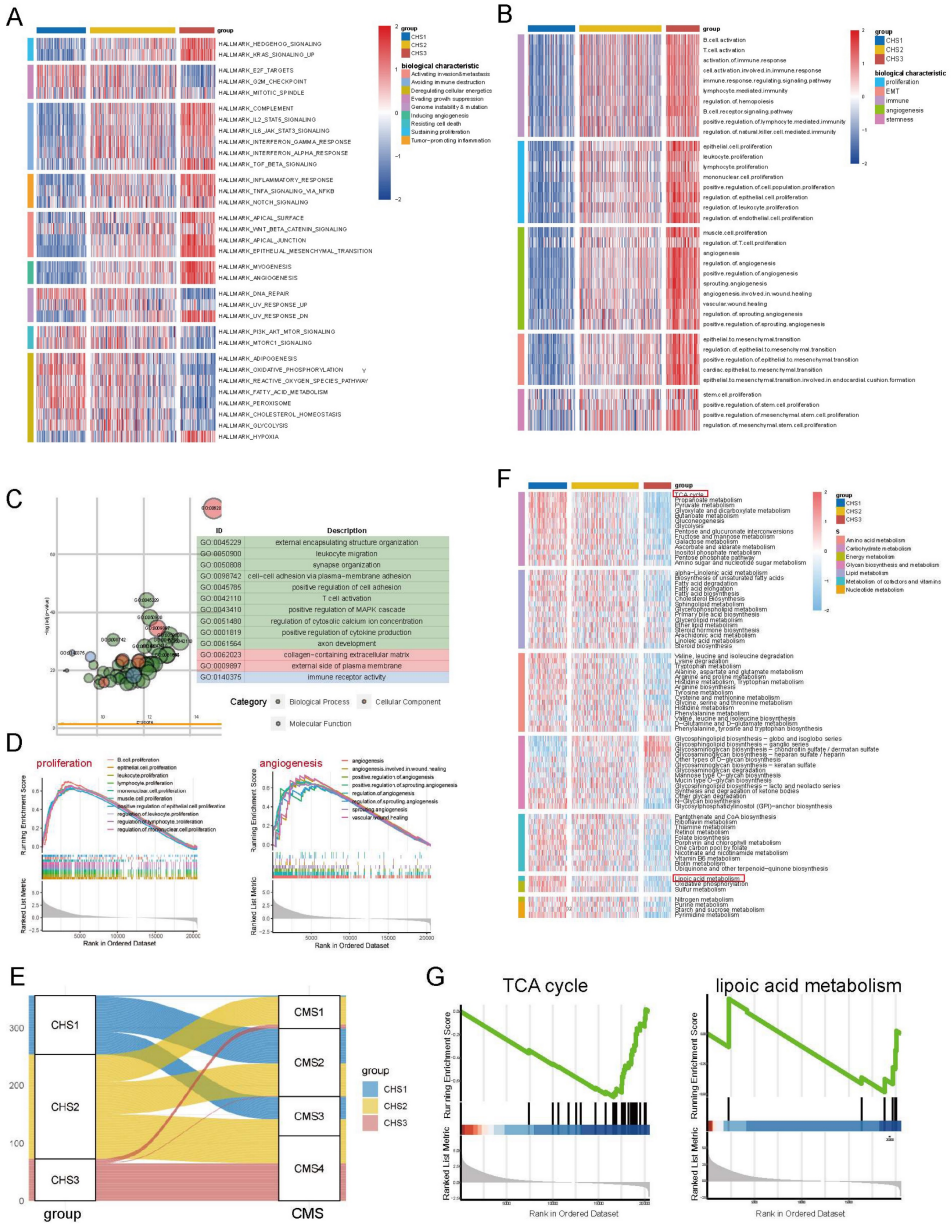
** Representative biological characteristics in CHSs.** A. The features of hallmarks of each subtype. B. The key biological traits of each subtype. C. The results of GO enrichment analysis on DEGs. D. The results of GSEA analysis on proliferation, angiogenesis. E. The correspondence between CHS and CMS. F. The metabolic profile among 3 CHSs. G. GSEA analysis of TCA cycle and lipoic acid metabolism.

We performed GSVA based on hallmarks 30 to profile the features of the CHSs. Surprisingly, there were significant differences among almost all gene sets among the subtypes (Figure 3A). The ability of cell proliferation, invasion, and metastasis, avoiding immune destruction, and inducing angiogenesis were stronger in CHS3, whereas evading growth suppression and dysregulation of energy metabolism were stronger in CHS1. The same trend was also observed in the three validation sets (Figure S2A-C). We performed GSVA based on GO genesets to further analyze multiple general biological traits. As shown in Figure 3B, there were disparities in proliferation, angiogenesis, invasion, stemness, and immunity, all of which were more conspicuous between CHS1 and CHS3.

To explore the differences between CHS1 and CHS3, we first performed a differential analysis to identify DEGs, based on which we performed GO analysis. The results showed that DEGs were mainly enriched in metastasis, and immune-related pathways (Figure 3C). Further, we observed that GSEA of representative pathways with classic biological traits were more active in CHS3 (Figure 3D, Figure S2G,I), which also verified the results of GSVA (Figure 3B). In the three validation cohorts, all the above results presented identical tendencies to the training sets (Figure S2D-F, Figure S3A-E).

The consensus molecular subtype (CMS) is probably the most robust classification for CRC. It categorized patients into 4 clusters, namely, CMS1 with hypermutation and immune activation; CMS2 with marked WNT and MYC signaling activation; CMS3, evident metabolic dysregulation; and CMS4 with prominent TGF-β activation, stromal invasion angiogenesis and poor prognosis 31. As shown in Figure 3E, CHS1 mainly corresponded to CMS2 and CMS3, CHS2 had no tendentious distribution, and CHS3 predominantly belonged to CMS4, which aligned with the hallmark features and prognostic characteristics of CHSs. The correspondence of both taxonomies was identical in the validation cohorts (Figure S3F).

### The metabolic profile of the CHSs

Since cell metabolism is associated with both cuproptosis and hypoxia 32,33, as well as the implications of energy metabolism in hallmark analyses, we performed GSVA based on all KEGG metabolic pathways to explore the metabolic profile of CHSs. This demonstrated that, except for glycan, the metabolism of other substances was the most active in CHS1. Notably, the TCA cycle and lipoic acid metabolism, closely related to cuproptosis, were much more active in CHS1 (Figure 3F, 3G), which corresponds to the physiological relationship between them and cuproptosis. Due to oxidative phosphorylation being a critical step in aerobic respiration following the TCA cycle, we also conducted GSEA on oxidative phosphorylation. The results indicated that there was no significant difference in the extent of oxidative phosphorylation between the CHS1 and CHS3 (*p* = 0.705). It is consistent with the findings from Tsvetkov et al., indicating that the impact of copper on cuproptosis is exerted through the TCA cycle rather than directly targeting the electron transport chain. The metabolic analysis results of the three validation datasets were similar to those of the training group (Figure S4A-E).

### Immune landscape among the CHSs

**Figure 4:**
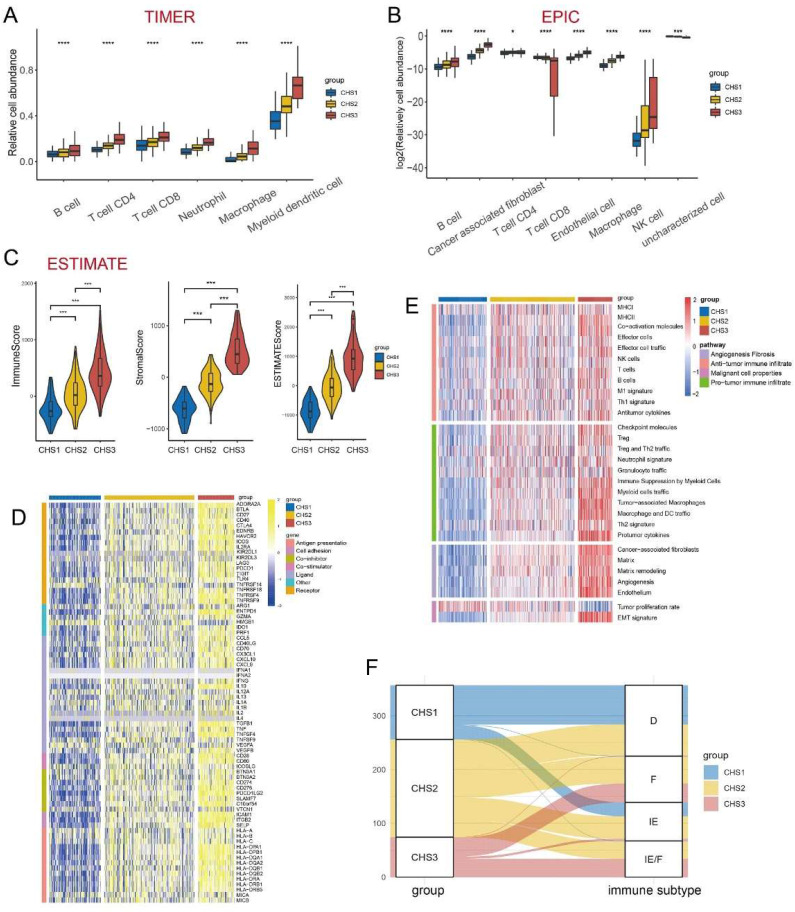
** The immune landscape among 3 CHSs.** A,B. The abundance of immune cells by TIMER, EPIC. C. The scores of Estimate. ∗, *p<*0.05; ∗∗, *p<*0.01; ∗∗∗, *p<*0.001. D. The expression of ICB in each subtype. ICB, immune checkpoint blockade. E. The evaluation of Fges for immune. Fges, functional gene expression signatures. F. The correspondence between CHS and Fges classification. *D*, immune-depleted; *F*, fibrotic; *IE*, immune-enriched, non-fibrotic; *IE/F*, immune-enriched, fibrotic.

The immune system acts as an important anti-tumor barrier 34. The abundance and diversity of the T-cell repertoire (TCR) and B-cell repertoire (BCR) were higher in CHS3, suggesting more infiltration of T and B cells in CHS3 (Figure S5A,B). The results of TIMER showed that six major immune cell clusters differed significantly among CHSs and were the most abundant in CHS3 (Figure 4A). The results of EPIC were largely consistent with TIMER. Notably, the fraction of cancer-associated fibroblasts (CAFs) was also much higher in CSH3 (Figure 4B), consistent with hallmarks and CMS results 35. The results of MCP-counter were also in accordance with TIMER and EPIC (Figure S5D).

Next, we used the ESTIMATE algorithm to assess the overall status of the TME (Figure 4C). The three indices obtained from ESTIMATE were highest in CSH3, suggesting that the immune cells and stromal components in CHS3 were relatively more abundant. The tumor purity of CHS3 was the lowest, which was negative with the estimated score. The scores of the validation cohorts presented the same trend (Figure S5E-G).

Mellman et al. summarized the complex anti-tumor process of immune cells as a cancer-immunity cycle 36, 37. Our analysis of the cancer-immunity cycle demonstrated that the release of cancer antigens and the infiltration of various immune cells were more enriched in CHS3. However, the recognition and killing of cancer cells appeared more inert in CHS3 (Figure S5H). The infiltration of more suppressive cells and the inhibitory effect of the TME in CHS3 might have caused this. Further, the validation datasets showed a similar tendency (Figure S5I).

We measured the expression of immune checkpoint blockade (ICB)-related molecules and observed that their expression was the highest in CHS3 (Figure 4D). A high lymphocyte fraction was also associated with better efficacy of ICB 38-40. The analysis demonstrated that the lymphocyte fraction was also the highest in CHS3 (Figure S5C). The efficacy of ICB is directly influenced by the TME 41. Fowler et al. established Fges (functional gene expression signatures) to classify the TME into four distinct subtypes 42. The analysis showed that the features of Fges in the three subtypes were distinct (Figure 4E). CHS1 mainly belonged to subtypes D and IE, and CHS3 mainly belonged to subtypes IE/F and F, indicating that CHS3 was highly fibrotic and responded poorly to immunotherapy (Figure 4F). The expression of ICB and the TME subtypes in the three validation cohorts presented the same tendency (Figure S6A-C). Therefore, improving the fibrotic stroma is likely a prerequisite for the satisfactory effect of immunotherapy in CHS3.

### Examining the characteristics of the subtypes at the single cell level

**Figure 5:**
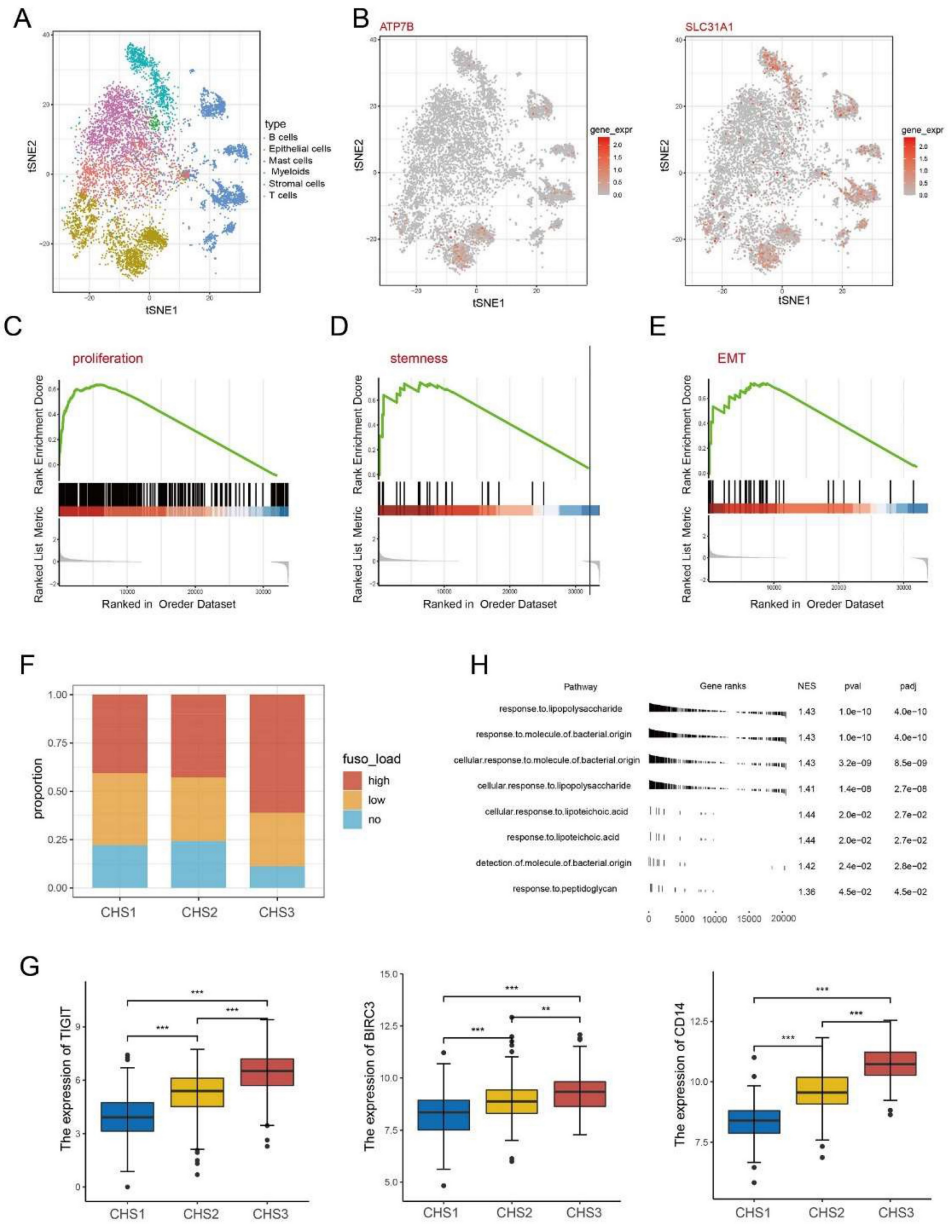
** Single cell and Fusobacterium nucleatum analysis.** A. The distribution of cell type in GSE144735. B. The expression of ATP7B and SLC31A1 in single cells. C-E. The GSEA analysis on proliferation, stemness, invasion of cancer cells. F. The abundance of F.n among 3 subtypes. H. The result of GSEA analysis on bacteria-related pathways. G. The expression of TIGIT, BIRC3, CD14 among 3 subtypes. ∗, *p<*0.05; ∗∗, *p<*0.01; ∗∗∗, *p<*0.001.

After annotating the cell types, we obtained 2,212 tumor cells from the single-cell dataset (Figure 5A). The expression of some key genes showed obvious disparities. For instance, the cuproptosis regulators ATP7B and SLC31A1 are almost exclusively expressed in tumor cells. In contrast, some genes such as SLC2A3 and RORA are highly expressed in other cells except tumor cells (Figure 5B, Figure S7A).

Our classifier classified the tumor cells into three clusters to observe the biological characteristics (Figure S7B) and verify the conclusions of the bulk transcriptomic data. Namely, the TCA cycle and lipoic acid metabolism were more active in CHS1 than in CHS3 (Figure S7C). Other representative features were stronger in CHS3 cells than in CHS1 (Figure 5C-E).

### DNA methylation pattern and features of the microbiota among the CHSs

Aberrant methylation is associated with tumorigenesis, apoptosis, and therapeutic resistance 43-45. The comparison of methylation sites between CHS1 and CHS3 revealed differentially methylated sites (Figure S7D). Immunity and lipid metabolism were among the top ten pathways of enrichment analysis based on differential sites (Figure S7E), which verified the reliability of the transcriptome analysis results from another dimension.

The importance of microbiota in the origin, development, and therapeutic response of CRC has been gradually recognized 46, especially *Fusobacterium nucleatum (F.n)* 47. The* F.n* abundance in CHS3 was significantly higher (Figure 5F). *TIGIT* and *BIRC3* are key factors interacting with *F.n* to suppress tumor immunity and promote chemoresistance in CRC 48, 49. LBP, MD2, TLR4 and CD14 are responsible for general bacterial effects 50. Analysis of the above genes revealed that their expression in CHS3 was significantly higher, consistent with the richness of *F.n* (Figure 5G, Figure S7F). GSEA based on the genesets of response to bacteria showed that the *p*-value of each pathway was less than 0.05 (Figure 5H). This indicates that *F.n* in tissues may alter the transduction of multiple signaling pathways, thereby affecting the tumorigenesis and progression of CRC.

### Construction of the competing endogenous RNA (ceRNA) network

Salmena et al. proposed the concept of ceRNAs to describe sophisticated regulatory relationships among different types of ncRNAs 51. It was recently discovered that they are non-negligible in various diseases, including CRC 52, 53. We performed differential analysis for miRNAs and obtained 150 differentially expressed miRNAs. Enrichment analysis demonstrated that differential miRNAs were enriched in JNK cascade, and interferon-gamma binding (Figure S8A). Based on the differential miRNAs and lncRNAs, we acquired 10 hub lncRNAs and 28 hub miRNAs using StarBase. Next, we utilized miRNA-mRNA predictive websites obtained 409 targeted mRNAs, and successfully visualized the ceNetwork with Cytoscope (Table S3, Figure S8B,C).

### Pan-cancer analysis

**Figure 6:**
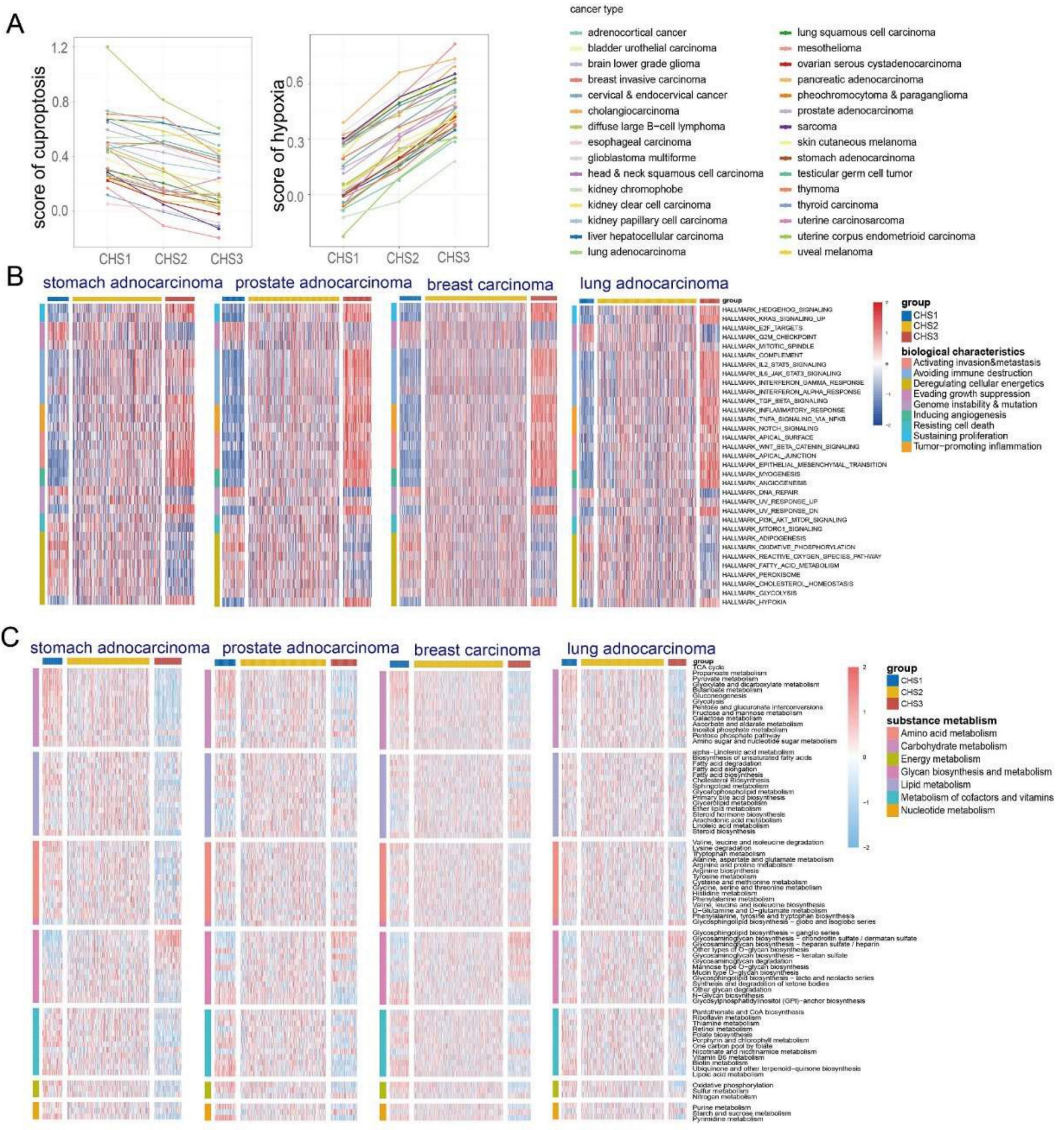
** Classic biological characteristics of CHSs in pan-cancer.** A. The characteristic of cuproptosis and hypoxia among CHSs in pan-cancer. B. The features of hallmarks of each subtype in representative cancer. C. The metabolic profile among CHSs in representative cancer.

Given that both cuproptosis and hypoxia are non-cancer specific, we explored whether the above-mentioned taxonomy is applicable to other cancers. The KNN was applied to classify cancers using TCGA-COADREAD as the training set. Most tumors had the same score characteristics as CRC—the cuproptosis score was highest in CHS1 and lowest in CHS3, whereas the hypoxia score was the opposite (Figure 6A).

#### Profile of pan-cancer hallmarks

**Figure 7:**
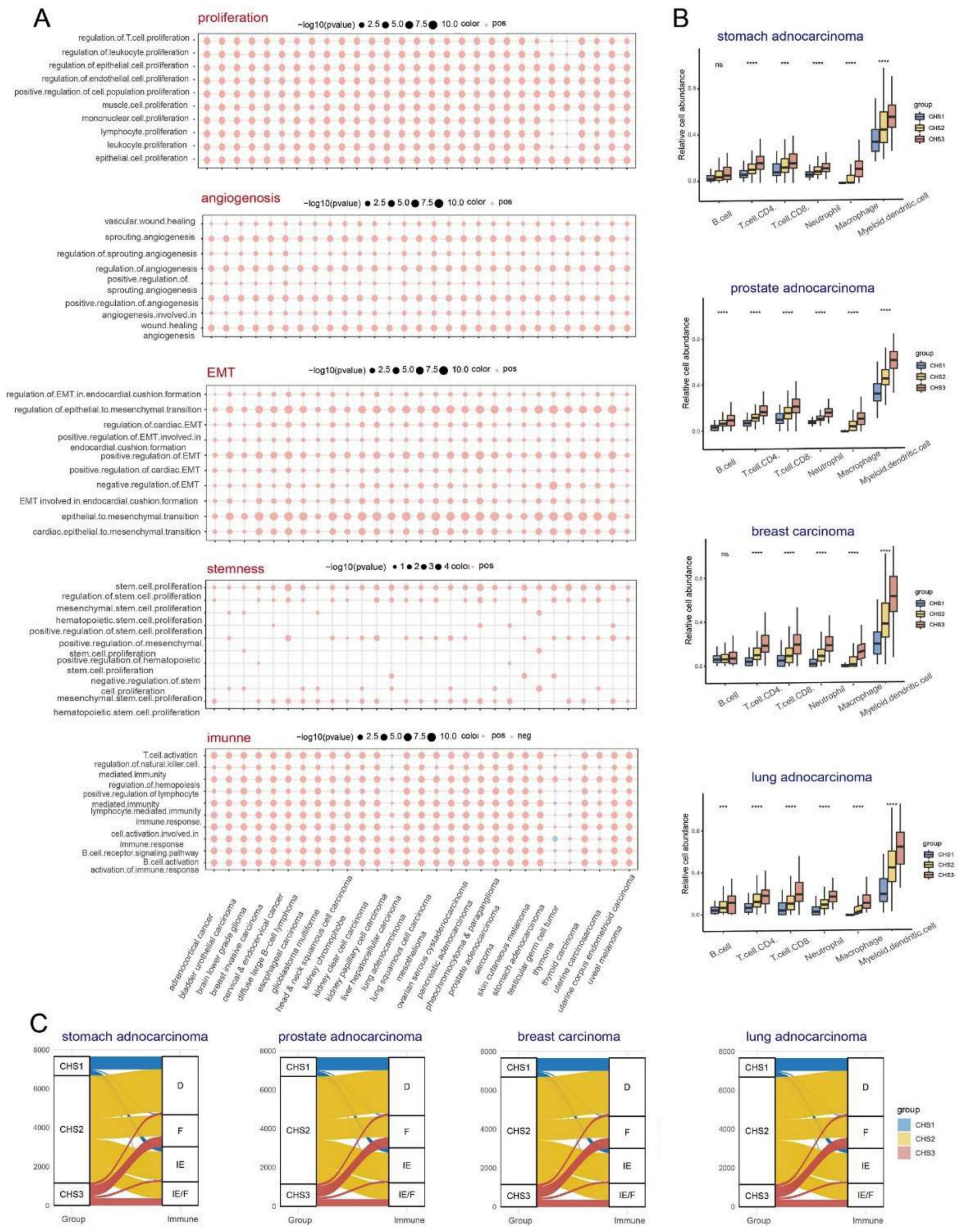
** The immune landscape among 3 CHSs in pan-cancer.** A. The results of GSEA analysis on proliferation, angiogenesis, invasion, stemness, and immune in pan-cancer. B. The abundance of immune cells by TIMER in representative cancer. ∗, *p<*0.05; ∗∗, *p<*0.01; ∗∗∗, *p<*0.001. C. The correspondence between CHS and Fges classification in representative cancer.

Since the hallmarks of CRC were significantly different among the CHSs, we performed GSVA of the hallmarks to investigate whether the CHSs of pan-cancer had the same biological features. In almost all cancers, CHS3 exhibited more active cell proliferation, metastasis, immune destruction, and angiogenesis, whereas CHS1 had stronger ability to evading growth inhibition and dysregulation of energy metabolism (Figure 6B, Figure S9). Further, we performed GSVA of various specific pathways in pan-cancer. Most cancers were identical to CRC, with significant differences in proliferation, stemness, invasion, angiogenesis, and immunity among the CHSs. The above differences were more evident between CHS1 and CHS3 (Figure S10A-E). The results of the GSEA were in accordance with the GSVA, i.e., the mentioned features were more active in CHS3 (Figure 7A).

#### Metabolic characteristics of pan-cancer

GSVA based on the KEGG metabolic pathway presented that the metabolic characteristics of most tumors had the same trend as that of CRC—the substance metabolism was the most active in CHS1, except for glycan biosynthesis (Figure 6C, Figure S11). The characteristics of cancers whose pathological type was adenocarcinoma had a higher similarity to CRC, highlighting the importance of tissue origin and pathological type. The activity of TCA cycle and lipoic acid metabolism presented the similar trend (Figure S12, S13). These results revealed that CHS classification is an important aspect of tumor metabolism, especially adenocarcinoma.

#### The immune landscape among pan-cancer CHSs

TIMER, EPIC, and MCP-counter were used to assess the pan-cancer immune cell infiltration. We found conspicuous differences among the CHSs for the six immune cells in most cancer types with TIMER and the trend was the same as in CRC (Figure 7B). The results obtained via EPIC and MCP-counter were also identical to those of CRC (Figure S14A,B; only representative cancer types are shown). The abundance and diversity of TCR and BCR showed the same trend (Figure S15A-D). According to the ESTIMATE assessment, the infiltration of immune cells and stromal components in most cancers was similar to that in CRC among each subtype (Figure S14C). As in CRC, the expression of ICB-related molecules was highest in CHS, but lowest in CHS1 (Figure S15E). Then, we classified the TME of pan-cancer into four subtypes based on Fges and compared them with the CHSs, and the correspondence was accordant with CRC. Namely, the majority of CHS1 belonged to subtype D, and CHS3 belonged to subtype IE/F or F, which confirmed the high fibrosis of the TME in CHS3 (Figure 7C).

### Exploration of the clustering in clinical application

#### Sensitivity of classic drugs and organoid validation

**Figure 8:**
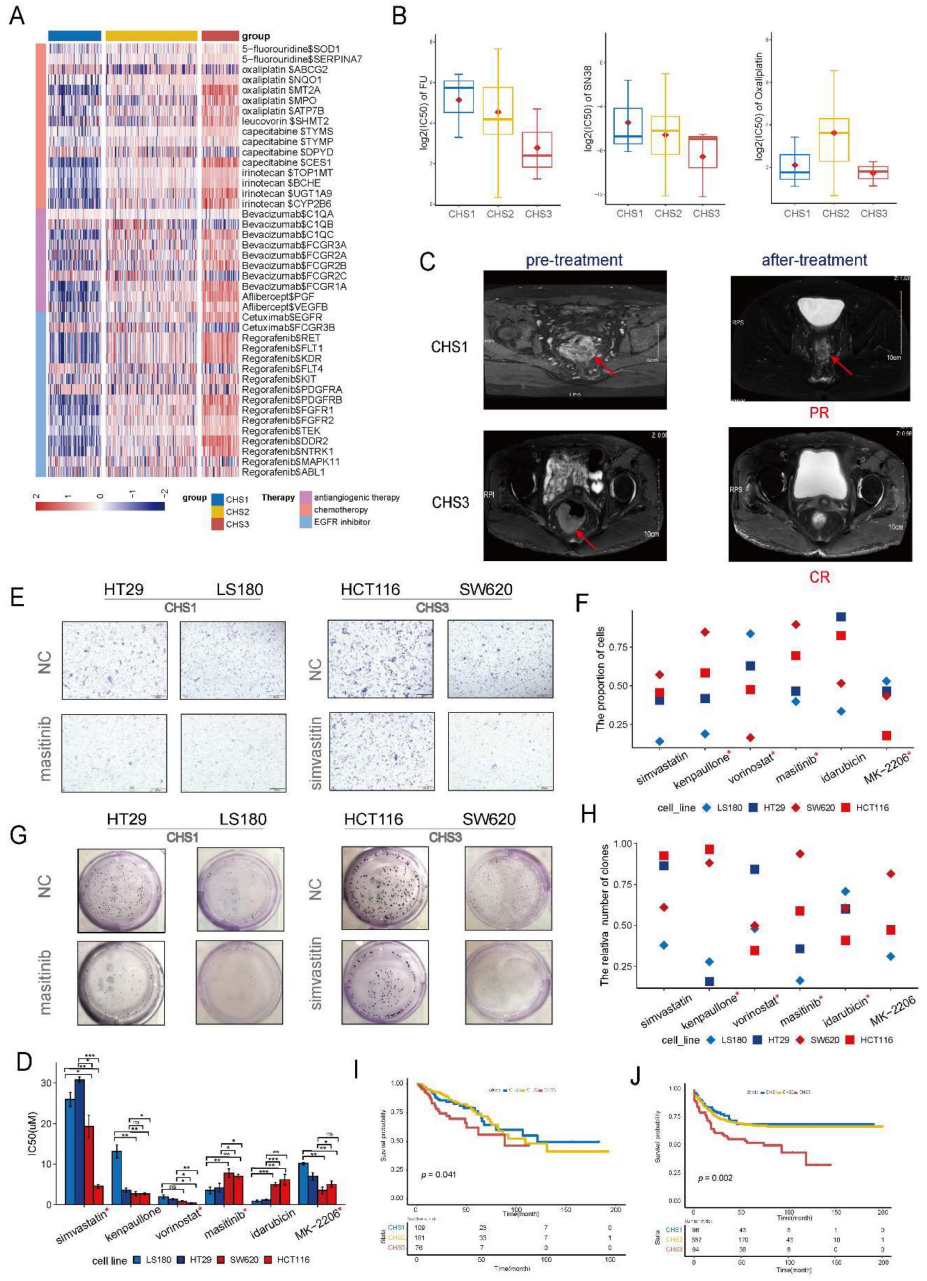
** Clinical application of the clustering.** A. The expression of targeted molecules of chemotherapeutic drugs. B. The IC50 of fluorine, SN38 and oxaliplatin in organoids. C. The MRI images of patients belonged to CHS1 and CHS3. MRI, magnetic resonance imaging; PR, partial response; CR, complete response. D. The IC50 of the drugs for colorectal cells. *, the assay results were consistent with the prediction of the cMap database. ^#^, the unit is nm. E. The representative images of transwell assay. NC, normal control. F. The result of transwell assay for CRC cell lines with screened drugs. NC, normal control. G. The representative images of colony-formation. NC, normal control. H. The results of colony-formation for CRC cell lines with screened drugs. NC, normal control. I.J. Kaplan-Meier plot for 3 CHSs in training set and GSE39582, respectively.

To evaluate the drug sensitivity of CHSs, we retrieved the target molecules of CRC drugs from Drugbank and analyzed their expression specifically within the CHSs. We observed distinct differences in the expression of multiple targets of chemotherapeutic and targeted drugs in the CHSs (Figure 8A). The higher expression of targeted molecules in CHS3 indicated that the cluster probably benefited more from the drugs.

Samples of organoids were successfully classified into CHSs with transcriptomic data (Figure S16A). The IC50 values of organoids was consistent with the DrugBank's prediction. CHS3 was the most sensitive to fluorine and SN38 (the active form of irinotecan *in vivo*), whereas CHS1 was relatively insensitive (Figure 8B). Patients of CSH3 indeed benefited more from chemotherapy according to clinical efficacy (Figure 8C). The analysis of GSE28702 also showed that CHS3 had the highest response rate to FOLFOX (Figure S16B). Together, these results indicate that this taxonomy is valuable in guiding clinical medication.

#### Prediction of novel drugs and cell validation

Using the cMap database, we identified five potent drugs for CHS1 and CHS3, and analyzed the correlations between the drugs and key genes (Figure S16C). To test the efficacy, we treated CRC cell lines belonging to the different CHSs with the drugs, except for masoprocol, which was unavailable. The classification results for the cell lines are listed in Table S4. We selected LS180, HT29, and HCT116, SW620 cells to represent CHS1 and CHS3, respectively. The expression of representative key genes between CHS1 and CHS3 cells is differential at transcription levels and protein levels (Figure S17A-B). The IC50 results showed that most drugs had a strong cell-killing effect on CRC cells, except forskolin, pazopanib, and MK-0752 (Figure S18). Among the remaining drugs, most IC50s for cell lines were consistent with the predicted tendency-- the sensitive drugs for CHS3 had lower IC50 values in HCT116 and SW620, and the sensitive drugs for CHS1 showed the opposite trend (Figure 8D). The transwell assay revealed that most drugs had an effective and differential impact on the migration of the two cell subtypes (Figure 8E,F). The drugs sensitive to CHS3 weakened the migration ability of HCT116 and SW620 to a greater extent. Colony formation presented a similar trend (Figure 8G, H). Generally, masitinib had more obvious effects on the proliferation, migration, and survival of CHS1 cells, suggesting that masitinib might have an unexpected anti-tumor effect in CHS1. Similarly, simvastatin was administered to CHS3.

#### Prognostic value of the clustering

The survival analysis of the training set showed that the outcomes of the CHSs were significantly different. CHS1 had the best prognosis, whereas CHS3 had the worst (Figure 8I). The same survival trend was observed in the validation set (Figure 8J). It was also consistent with the indication of Braf mutation (Figure 2B).

We constructed a prognostic model based on the prognostic differences between CHS1 and CHS3. The details of the model were provided in Table S6. Patients with higher scores had a poorer prognosis than their counterparts (Figure S16D). In addition, we built a nomogram with the model for clinical application (Figure S16E). The areas under the curves of the time-dependent ROC curves at 1, 3, and 5 years were 0.8, 0.8, and 0.81, respectively (Figure S16F). Finally, we constructed calibration curves to test the consistency of the predictive survival probability and actual information (Figure S16G). Both the ROC and calibration curves validated the good predictive performance of the model.

## Discussion

A robust classifier is valuable for precision medicine and improving prognosis because of the heterogeneity and prognostic disparity of cancers. Since the unique mechanism of cuproptosis has been reported, several studies have investigated its correlation with multiple cancers 54-56. Considering the relationship between cuproptosis and hypoxia, it is important to explore their role in classifying CRC and pan-cancer to guide clinical application.

In this study, we identified three distinct subtypes based on cuproptosis and hypoxia using TCGA_COADREAD as the training set and validated it in single-cell and bulk validation sets. CHS1 is characterized by high sensitivity for cuproptosis, low degree of hypoxia, active metabolism, poor immune infiltration, less fibrosis in the TME, and the best prognosis among the CHSs. In contrast, CHS3 is characterized by low sensitivity for cuproptosis, high degree of hypoxia, relatively inactive metabolism, abundant immune infiltration, more fibrosis in the TME, and poor prognosis. The characteristics of CHS2 are intermediate. Heterogeneity is a fundamental feature of cancers 57. Thus, we applied this classification to pan-cancers and surprisingly found that it enabled to classify most samples into three distinct clusters.

The results indicated that CHS1 might be more sensitive to cuproptosis inducers. CHS3 of CRC might have a higher response rate to classic drugs, according to the analysis of transcriptomics and organoids. Although hypoxia is responsible for resistance to several chemotherapeutic drugs 57, 58, and confers tumor cells the ability to activate pleiotropic mechanisms for survival 9. The good response of CHS3 might be due to the much higher expression of targeted genes, although their degree of hypoxia is more severe. Combining the results from databases and assays, masitinib and simvastatin might have specific effects on CHS1 and CHS3 in CRC, respectively. As a predominant microbiota in CRC, *F.n* influences tumorigenesis, proliferation, angiogenesis, and chemoresistance of CRC by affecting TME and multiple signaling pathways 60,61. The analysis of *F.n* demonstrated that its differential richness might be responsible for the differences among CHSs. Additionally, we constructed a prognostic model and a ceRNA network for CRC to provide a reference for prognostic prediction and ncRNA research.

Cuproptosis and hypoxia are closely related to metabolism 5, 62, and our study demonstrated that the metabolism of most substances, including the TCA cycle and lipoic acid, was more active in CHS1. The TCA cycle is pivotal for aerobic respiration 63. Severe hypoxia accompanying lower TCA cycle is consistent with the physiology. Analysis of the hallmarks indicated a stronger malignancy in CHS3. The insensitivity of CHS3 to cuproptosis may allow higher Cu levels in physiological state, which creates a favorable niche for proliferation, metastasis and angiogenesis 64-66. Moreover, hypoxia promotes a bias toward metastasis and stemness 67, 68. Therefore, we speculate that low sensitivity for cuproptosis and high-degree hypoxia may synergistically account for the active proliferation and metastasis in CHS3.

Prior researches have shown a close relationship between cuproptosis and tumor immunity 55, 69, 70. Alterations in the immune profile always occur when tumors experience hypoxia, such as T-cell dysfunction, tumor-associated macrophages recruitment, and increased ICB resistance 71 - 73. Our analysis of TME revealed that immune infiltration in CHS3 was the most abundant, including anti-tumor cells, MDSCs, and CAFs. Given the impactful immunosuppressive function of MDSCs and CAF 74, even if the immune infiltration of CHS3 was more abundant, the function of tumor suppression and immune killing was not necessarily superior to that of the other two subtypes.

As a major type of immunotherapy, ICBs have provided hope to patients once considered incurable. Multiple ICB drugs have been approved by the FDA 75. The highest level of ICB expression in CHS3 indicates the potential for ICB therapy in these patients. Also, the fibrosis of TME in CHS3 was the highest. Therefore, we assume that the high infiltration of immunosuppressive cells, high fibrosis of the TME and high ICB expression inhibited immune surveillance and killing in CSH3, leading to poor prognosis. Bagaev et al. revealed that the more severe fibrosis of the TME, the worse ICB effect. Given the adverse effects of fibrosis and hypoxia on ICB treatment 71, 76, improving fibrosis and hypoxia in the TME should be a prerequisite for the maximum efficacy of ICB in CHS3. Anti-TGF-β is one of general approaches to improve fibrosis in the TME 77. TGF-β expression greatly increases under severe hypoxic condition 78. It plays a vital role in cancers under hypoxia, actively participating in the regulation of TME. The TGF-β signaling synergizes with hypoxia to exert profound effects on extracellular matrix remodeling, promote tumor bone metastases, and modulate EMT in various cancers 79. Considering the synergy of hypoxia and TGF-β, combining anti-TGF-β drugs (e.g., vactosertib) and immunotherapy probably achieves surprising effects in CHS3.

Although our study provides a robust classification algorithm based on cuproptosis and hypoxia with a multi-omic analysis of CRC, which is applicable to pan-cancer, two major drawbacks still require further exploration. First, survival differences between the three subtypes for most tumors in pan-cancer were not as significant as those in CRC, suggesting that more factors must be considered for predicting their prognosis. Second, the treatment of pan-cancers was not explored due to incomplete information.

In conclusion, we performed a multi-omic analysis of CRC based on cuproptosis and hypoxia, classifying patients into three well-characterized subtypes. The subtyping algorithm is applicable to pan-cancers, and the general biological processes and immune profiles of each CHS are similar in pancancers. For CRC, chemotherapy and targeted therapy are more effective in CHS3, and improving hypoxia may further promote these effects. CHS3 may benefit greatly from ICB therapy under anti-fibrosis and anti-hypoxia conditions for most pan-cancer cases.

## Supplementary Material

Supplementary figures and tables.

## Figures and Tables

**Table 1:** The profile of datasets

	Dataset	Sample Type	Number	Source
**Training set**	TCGA_COADREAD	tissue	381	TCGA
**Validation set**	GSE39582	tissue	566	GEO
	GSE196576	tissue	578	GEO
	GSE28702	tissue	83	GEO
	GSE144735	single cell	8254	GEO
	TCGA_PANCAN*	tissue	9185	TCGA
**Re-validation set**	-	organoid	28	this study
	-	cell line	4	this study

* TCGA_PANCAN, including Adrenocortical carcinoma (ACC), Bladder urothelial carcinoma (BLCA), Breast invasive carcinoma (BRCA), Cervical squamous cell carcinoma and Endocervical adenocarcinoma (CESC), Cholangiocarcinoma (CHOL), Colon adenocarcinoma/Rectum adenocarcinoma (COADREAD), Diffuse large B-cell lymphoma (DLBL), Esophageal carcinoma (ESCA), Stomach adenocarcinoma (STAD), Glioma (GBMLGG), Head and Neck squamous cell carcinoma (HNSC), Kidney chromophobe (KICH), Kidney renal clear cell carcinoma (KIRC), Kidney renal papillary cell carcinoma (KIRP), Liver hepatocellular carcinoma (LIHC), Lung adenocarcinoma (LUAD), Lung squamous cell carcinoma (LUSC), Mesothelioma, Ovarian serous cystadenocarcinoma (OV), Pancreatic adenocarcinoma (PAAD), Pheochromocytoma and Paraganglioma (PCPG), Prostate adenocarcinoma (PRAD), Sarcoma, Skin cutaneous melanoma (SKCM), Testicular germ cell tumor (TGCT), Thyroid carcinoma (THCA), Uterine Corpus Endometrial carcinoma (UCEC), Uterine carcinosarcoma (UCS), and Uveal melanoma (UVM).

## Abbreviations

Cu: copper
PCD: programmed cell death
TCA: tricarboxylic acid
CRC: colorectal cancer
TME: tumor microenvironment
DMEM: Dulbecco's modified eagle's medium
FBS: fetal bovine serum
MSigDB: Molecular Signatures Database
GO: Gene ontology
GSEA: gene set enrichment analysis
GSVA: gene set variation analysis
KEGG: Kyoto Encyclopedia of Genes and Genomes
MCP-counter: microenvironment cell-populations counter
ncRNA: non-coding RNA
CHS: cuproptosis-hypoxia subtype
CRCO: colorectal cancer organoid
DEG: differential expression gene
ROC: receiver operating characteristic
SNP: single nucleotide polymorphism
CMS: consensus molecular subtype
TCR: T-cell repertoire
BCR: B-cell repertoire
CAF: cancer-associated fibroblast
ICB: immune checkpoint blockade
*F.n*: *Fusobacterium nucleatum*
ceRNA: competing endogenous RNA
MDSC: myeloid-derived suppressor cell
