# Supplementary material for: Unraveling Colorectal Cancer and Pan-cancer Immune Heterogeneity and Synthetic Therapy Response Using Cuproptosis and Hypoxia Regulators by Multi-omic Analysis and Experimental Validation
Source: Int J Biol Sci. 2023 Jul 9;19(11):3526–43. doi: 10.7150/ijbs.84781 (PMC10367564; doi:10.7150/ijbs.84781)

Supplementary Figures

Figure S1. Clinical characteristics of training set and scores characteristics of validation sets.

A. The classification based on unsupervised clustering.

B. The feature of cuproptosis and hypoxia among 3 CHSs.

C. The T ,N, M stage of 3 CHSs in training set.

D. The age of 3 CHSs in training set.

E. The proportion of gender in 3 CHSs in training set.

F. The feature of cuproptosis and hypoxia in 3 validation sets.

G. The situation of SNP types in 3 subtypes.

Figure S2. Representative biological characteristics in 3 CHSs.

A-C. The features of hallmarker of each subtype in GSE196576,GSE28702 and GSE39582.

D-F. The key biological traits of each subtype in GSE196576,GSE28702 and GSE39582.

G-I. The results of GSEA analysis on invasion, stemness, and immune in trainning set.

Figure S3. Continued Figureure to FigureS2

A-E. The results of GSEA analysis on proliferation, angiogenesis, invasion, stemness, and immune in 3 validation datasets.

F. The correspondence between CHS and CMS in 3 validation datasets.

Figure S4. The metabolic characteristics in validation datasets.

A-C. The features of metabolic pathways of each subtype in 3 validation datasets.

D. GSEA analysis of TCA cycle metabolism in 3 validation datasets.

E. GSEA analysis of lipoic acid in 3 validation datasets.

Figure S5. The immune-related features.

A. The abundance and diversity of TCR .

∗, $P<0.05$; ∗∗, $P<0.01$; ∗∗∗, $P<0.001$. Wilcoxon test was applied.

B. The abundance and diversity of BCR .

∗, $P<0.05$; ∗∗, $P<0.01$; ∗∗∗, $P<0.001$. Wilcoxon test was applied.

C. Lymphocyte fraction in CHSs.

∗, $P<0.05$; ∗∗, $P<0.01$; ∗∗∗, $P<0.001$. Wilcoxon test was applied.

D. The abundance of immune cells by MCP-counter.

∗, $P<0.05$; ∗∗, $P<0.01$; ∗∗∗, $P<0.001$. Anova test was applied.

E-G. The scores of Estimate in 3 validation datasets.

∗, $P<0.05$; ∗∗, $P<0.01$; ∗∗∗, $P<0.001$. Anova test was applied.

H.I. The status of cancer-immunity cycle in trainning set and validation datasets.

∗, $P<0.05$; ∗∗, $P<0.01$; ∗∗∗, $P<0.001$. Anova test was applied.

Figure S6. Continued Figureure to FigureureS5.
A. The expression of ICB in validation datasets.
B. The evaluation of functional gene expression signatures (Fges) for immune in validation datasets.
C. The correspondence between CHS and Fges classification in validation datasets.

Figure S7. The related analysis of multi-omics.
A. The expression of SLC2A3 and RORA n single cells.
B. The feature of cuproptosis and hypoxia among CHSs of single cells.
C. GSEA analysis of TCA cycle and lipoic acid metabolism in single cells.
D. The vocalno plot of differential methylation sites between CHS1 and CHS3.
F. The expression of LBP, LY96, TLR4. *, P<0.05; **, P<0.01; ***, P<0.001.

Figure S8. The construction of ceRNA Network.
A. The GO enrichment analysis of differential miRNAs.
B. The workflow of constructing the ceRNA Network.
C. The ceRNA Network including 10 hub lncRNAs and 28 hub miRNAs.

Figure S9. The features of hallmarker of each subtype in pan-cancer.

Figure S10. The characteristic of biological features in representative cancer
A-E.The features of proliferation, angiogenesis, invasion, stemness and immune among CHSs.

Figure S11. The metabolic profile among CHSs in pan-cancer.

Figure S12. The GSEA of TCA in pancancers.

Figure S13. The GSEA of lipoic acid metabolism in pancancers.
Figure S14. The immune-related features in pan-cancer.
A. The abundance of immune cells by EPIC.
A. *, P<0.05; **, P<0.01; ***, P<0.001. Anova test was applied.
B. The abundance of immune cells by MCP-counter.
*, P<0.05; **, P<0.01; ***, P<0.001. Anova test was applied.
C. The scores of Estimate in representative cancer.
*, P<0.05; **, P<0.01; ***, P<0.001. Anova test was applied.

Figure S15. Continued Figureure to FigureS12
A-D. The abundance and diversity of TCR and BCR in stomach adenocarcinoma, lung adenocarcinoma, breast adenocarcinoma, prostate adenocarcinoma.
*, P<0.05; **, P<0.01; ***, P<0.001. Wilcoxon test was applied.

Figure S16. The results of assays and the prognostic signature.
A. The feature of cuproptosis and hypoxia among CHSs of organoid samples (number of CHS1 is 3, number of CHS2 is 20, number of CHS3 is 3).
B. The response rate of FOLFOX in each CHSs in GSE28702.
C. The sensitive drugs for CHS1 (lower left) and CHS3 (upper right) from the prediction of cMap, respectively.
D. Kaplan−Meier plot of two groups obtained from the prognostic signature. Log-rank test was applied.
E. The nomogram based on the prognostic signature.
F. The time-ROC curve of of nomogram at 1, 2, 3-year.
G. The calibration curves at 1, 2, 3-year.

Figure S17. The expression of representative key genes between CHS1 cells (HT29, LS180) and CHS3 cells (SW620, HCT116).
A. The results of qPCR.
B. The results of western blot.

Figure S18. The IC50 of screened drugs in HT29， LS180， HCT116， SW620， respectively.
A-F. The IC50 of masitinib, simvastatin, kenpaullone, vorinostat, MK-2206, idarubicin.

Figure S1

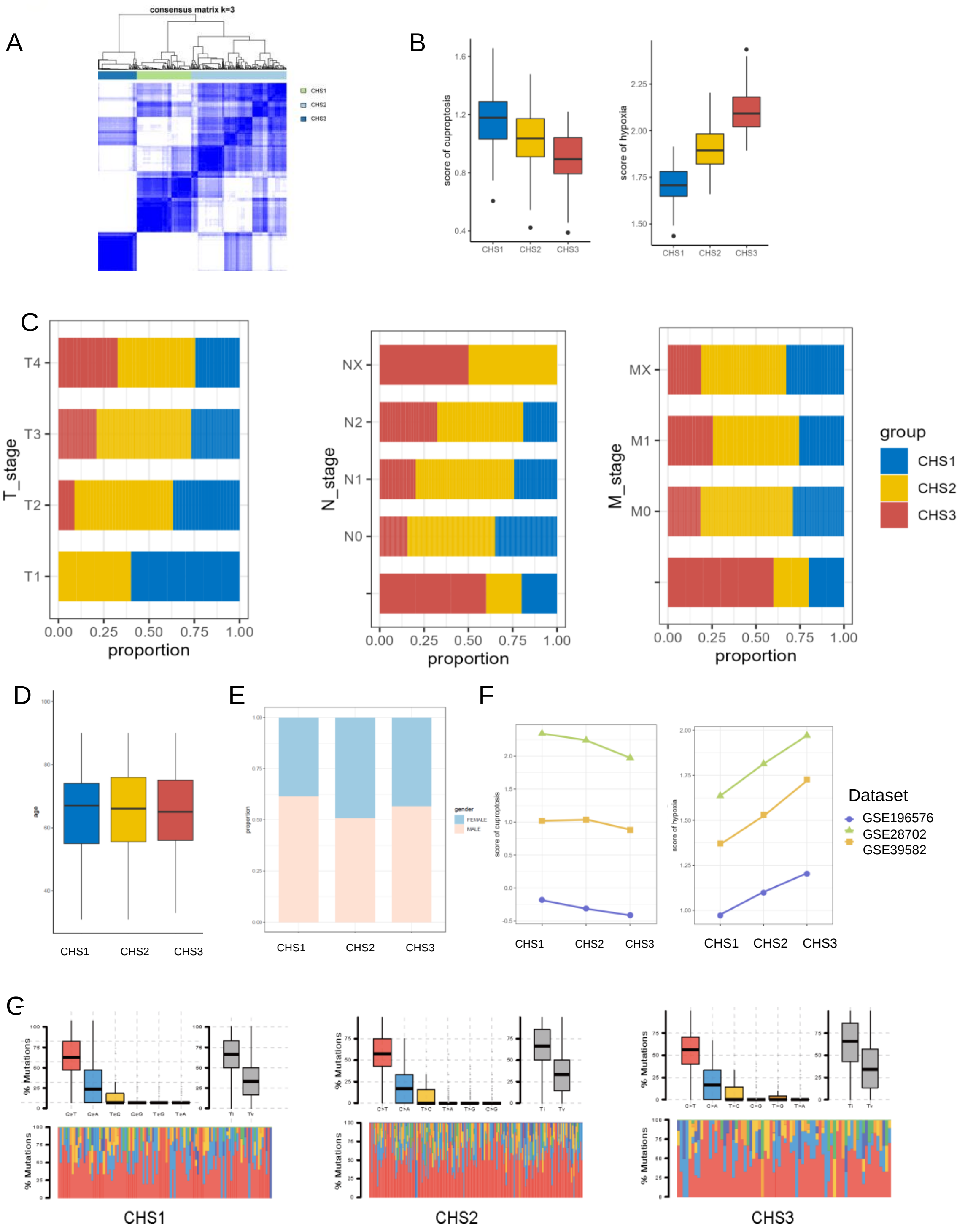

A
consensus matrix k=3
CHS1
CHS2
CHS3
B
score of cuproptosis
score of hypoxia
CHS1
CHS2
CHS3
C
T_stage
N_stage
M_stage
proportion
group
CHS1
CHS2
CHS3
D
age
E
proportion
gender
FEMALE
MALE
F
score of cuproptosis
score of hypoxia
Dataset
GSE196576
GSE28702
GSE39582
G
% Mutations
CHS1
CHS2
CHS3

Figure S2

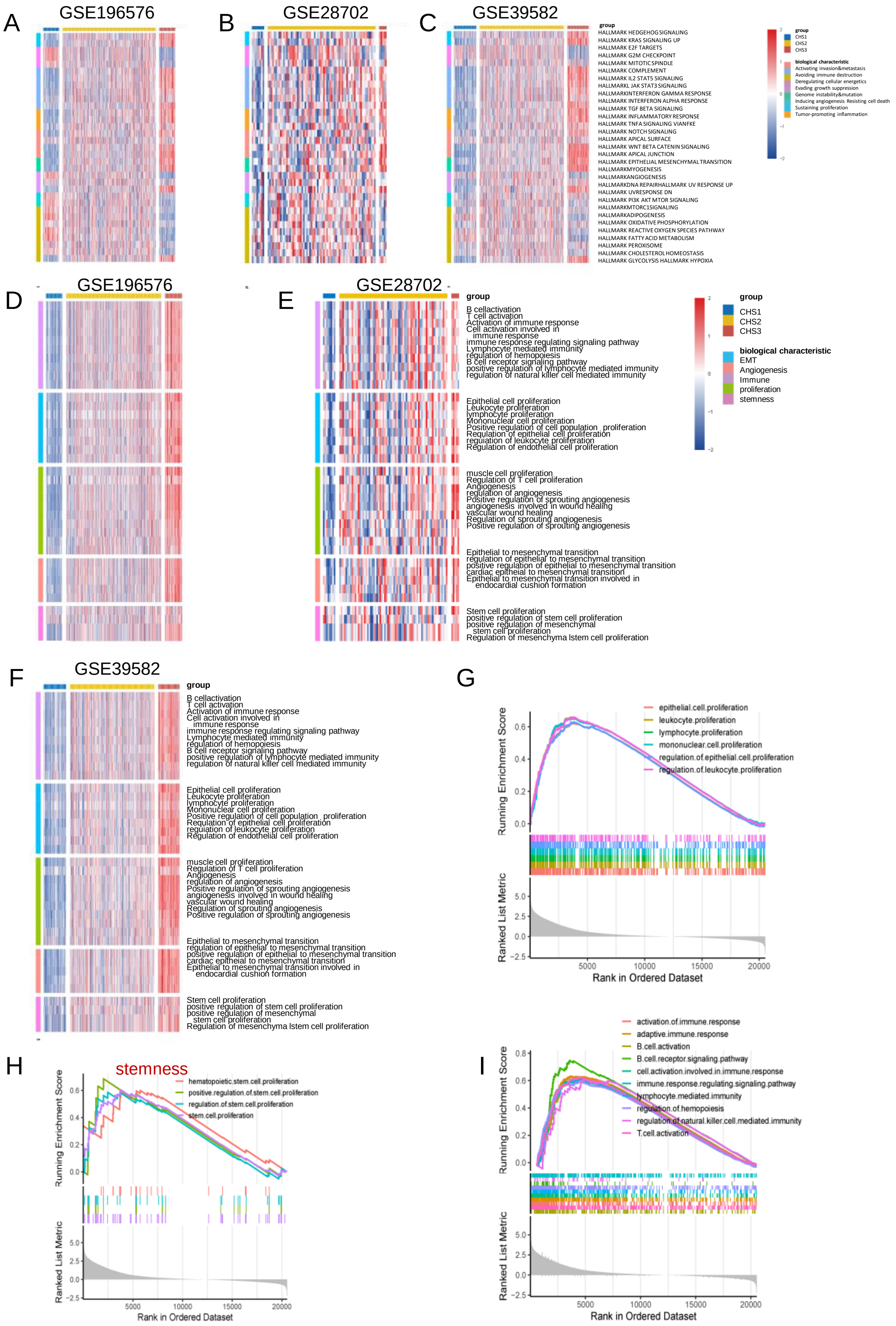

A
GSE196576
B
GSE28702
C
GSE39582
group
HALLMARK HEDGEHOG SIGNALING
HALLMARK KRAS SIGNALING UP
HALLMARK E2F TARGETS
HALLMARK G2M CHECKPOINT
HALLMARK MITOTIC SPINDLE
HALLMARK COMPLEMENT
HALLMARK IL2 STAT5 SIGNALING
HALLMARKL JAK STAT3 SIGNALING
HALLMARKINTERFERON GAMMA RESPONSE
HALLMARK INTERFERON ALPHA RESPONSE
HALLMARK TGF BETA SIGNALING
HALLMARK INFLAMMATORY RESPONSE
HALLMARK TNFA SIGNALING VIANFKE
HALLMARK NOTCH SIGNALING
HALLMARK APICAL SURFACE
HALLMARK WNT BETA CATENIN SIGNALING
HALLMARK APICAL JUNCTION
HALLMARK EPITHELIAL MESENCHYMAL TRANSITION
HALLMARKMYOGENESIS
HALLMARKANGIOGENESIS
HALLMARKDNA REPAIRHALLMARK UV RESPONSE UP
HALLMARK UVRESPONSE DN
HALLMARK PI3K AKT MTOR SIGNALING
HALLMARKMTORC1SIGNALING
HALLMARKADIPOGENESIS
HALLMARK OXIDATIVE PHOSPHORYLATION
HALLMARK REACTIVE OXYGEN SPECIES PATHWAY
HALLMARK FATTY ACID METABOLISM
HALLMARK PEROXISOME
HALLMARK CHOLESTEROL HOMEOSTASIS
HALLMARK GLYCOLYSIS HALLMARK HYPOXIA
group
CHS1
CHS2
CHS3
biological characteristic
Activating invasion&metastasis
Avoiding immune destruction
Deregulating cellular energetics
Evading growth suppression
Genome instability&mutation
Inducing angiogenesis Resisting cell death
Sustaining proliferation
Tumor-promoting inflammation
D
GSE196576
E
GSE28702
group
B cellactivation
T cell activation
Activation of immune response
Cell activation involved in immune response
immune response regulating signaling pathway
Lymphocyte mediated immunity
regulation of hemopoiesis
B cell receptor signaling pathway
positive regulation of lymphocyte mediated immunity
regulation of natural killer cell mediated immunity
Epithelial cell proliferation
Leukocyte proliferation
lymphocyte proliferation
Mononuclear cell proliferation
Positive regulation of cell population proliferation
Regulation of epithelial cell proliferation
regulation of leukocyte proliferation
Regulation of endothelial cell proliferation
muscle cell proliferation
Regulation of T cell proliferation
Angiogenesis
regulation of angiogenesis
Positive regulation of sprouting angiogenesis
angiogenesis involved in wound healing
vascular wound healing
Regulation of sprouting angiogenesis
Positive regulation of sprouting angiogenesis
Epithelial to mesenchymal transition
regulation of epithelial to mesenchymal transition
positive regulation of epithelial to mesenchymal transition
cardiac epitheial to mesenchymal transition
Epithelial to mesenchymal transition involved in endocardial cushion formation
Stem cell proliferation
positive regulation of stem cell proliferation
positive regulation of mesenchymal stem cell proliferation
Regulation of mesenchyma lstem cell proliferation
group
CHS1
CHS2
CHS3
biological characteristic
EMT
Angiogenesis
Immune
proliferation
stemness
F
GSE39582
G
epithelial.cell.proliferation
leukocyte.proliferation
lymphocyte.proliferation
mononuclear.cell.proliferation
regulation.of.epithelial.cell.proliferation
regulation.of.leukocyte.proliferation
Running Enrichment Score
Ranked List Metric
Rank in Ordered Dataset
H
stemness
hematopoietic.stem.cell.proliferation
positive.regulation.of.stem.cell.proliferation
regulation.of.stem.cell.proliferation
stem.cell.proliferation
Running Enrichment Score
Ranked List Metric
Rank in Ordered Dataset
I
activation.of.immune.response
adaptive.immune.response
B.cell.activation
B.cell.receptor.signaling.pathway
cell.activation.involved.in.immune.response
immune.response.regulating.signaling.pathway
lymphocyte.mediated.immunity
regulation.of.hemopoiesis
regulation.of.natural.killer.cell.mediated.immunity
T.cell.activation
Running Enrichment Score
Ranked List Metric
Rank in Ordered Dataset

Figure S3

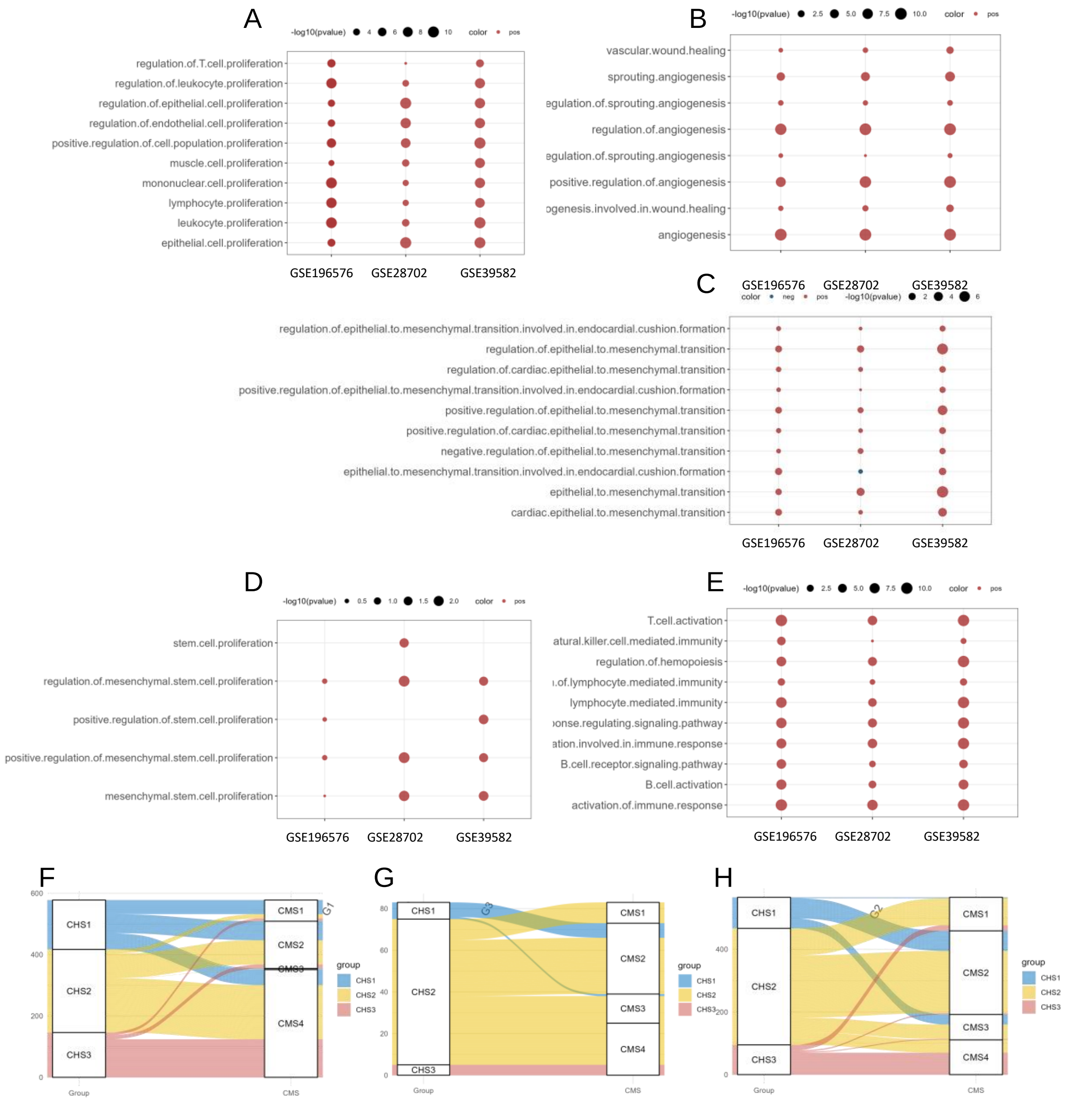

A
-log10(pvalue) 4 6 8 10 color pos
regulation.of.T.cell.proliferation
regulation.of.leukocyte.proliferation
regulation.of.epithelial.cell.proliferation
regulation.of.endothelial.cell.proliferation
positive.regulation.of.cell.population.proliferation
muscle.cell.proliferation
mononuclear.cell.proliferation
lymphocyte.proliferation
leukocyte.proliferation
epithelial.cell.proliferation
GSE196576 GSE28702 GSE39582
B
-log10(pvalue) 2.5 5.0 7.5 10.0 color pos
vascular.wound.healing
sprouting.angiogenesis
egulation.of.sprouting.angiogenesis
regulation.of.angiogenesis
egulation.of.sprouting.angiogenesis
positive.regulation.of.angiogenesis
ogenesis.involved.in.wound.healing
angiogenesis
GSE196576 GSE28702 GSE39582
C
color neg pos -log10(pvalue) 2 4 6
regulation.of.epithelial.to.mesenchymal.transition.involved.in.endocardial.cushion.formation
regulation.of.epithelial.to.mesenchymal.transition
regulation.of.cardiac.epithelial.to.mesenchymal.transition
positive.regulation.of.epithelial.to.mesenchymal.transition.involved.in.endocardial.cushion.formation
positive.regulation.of.epithelial.to.mesenchymal.transition
positive.regulation.of.cardiac.epithelial.to.mesenchymal.transition
negative.regulation.of.epithelial.to.mesenchymal.transition
epithelial.to.mesenchymal.transition.involved.in.endocardial.cushion.formation
epithelial.to.mesenchymal.transition
cardiac.epithelial.to.mesenchymal.transition
GSE196576 GSE28702 GSE39582
D
-log10(pvalue) 0.5 1.0 1.5 2.0 color pos
stem.cell.proliferation
regulation.of.mesenchymal.stem.cell.proliferation
positive.regulation.of.stem.cell.proliferation
positive.regulation.of.mesenchymal.stem.cell.proliferation
mesenchymal.stem.cell.proliferation
GSE196576 GSE28702 GSE39582
E
-log10(pvalue) 2.5 5.0 7.5 10.0 color pos
T.cell.activation
atural.killer.cell.mediated.immunity
regulation.of.hemopoiesis
ı.of.lymphocyte.mediated.immunity
lymphocyte.mediated.immunity
ɔonse.regulating.signaling.pathway
ation.involved.in.immune.response
B.cell.receptor.signaling.pathway
B.cell.activation
activation.of.immune.response
GSE196576 GSE28702 GSE39582
F
600 400 200 0
CHS1 CHS2 CHS3
CMS1 CMS2 CMS3 CMS4
G1
group CHS1 CHS2 CHS3
Group CMS
G
80 60 40 20 0
CHS1 CHS2 CHS3
CMS1 CMS2 CMS3 CMS4
G3
group CHS1 CHS2 CHS3
Group CMS
H
400 200 0
CHS1 CHS2 CHS3
CMS1 CMS2 CMS3 CMS4
G2
group CHS1 CHS2 CHS3
Group CMS

Figure S4

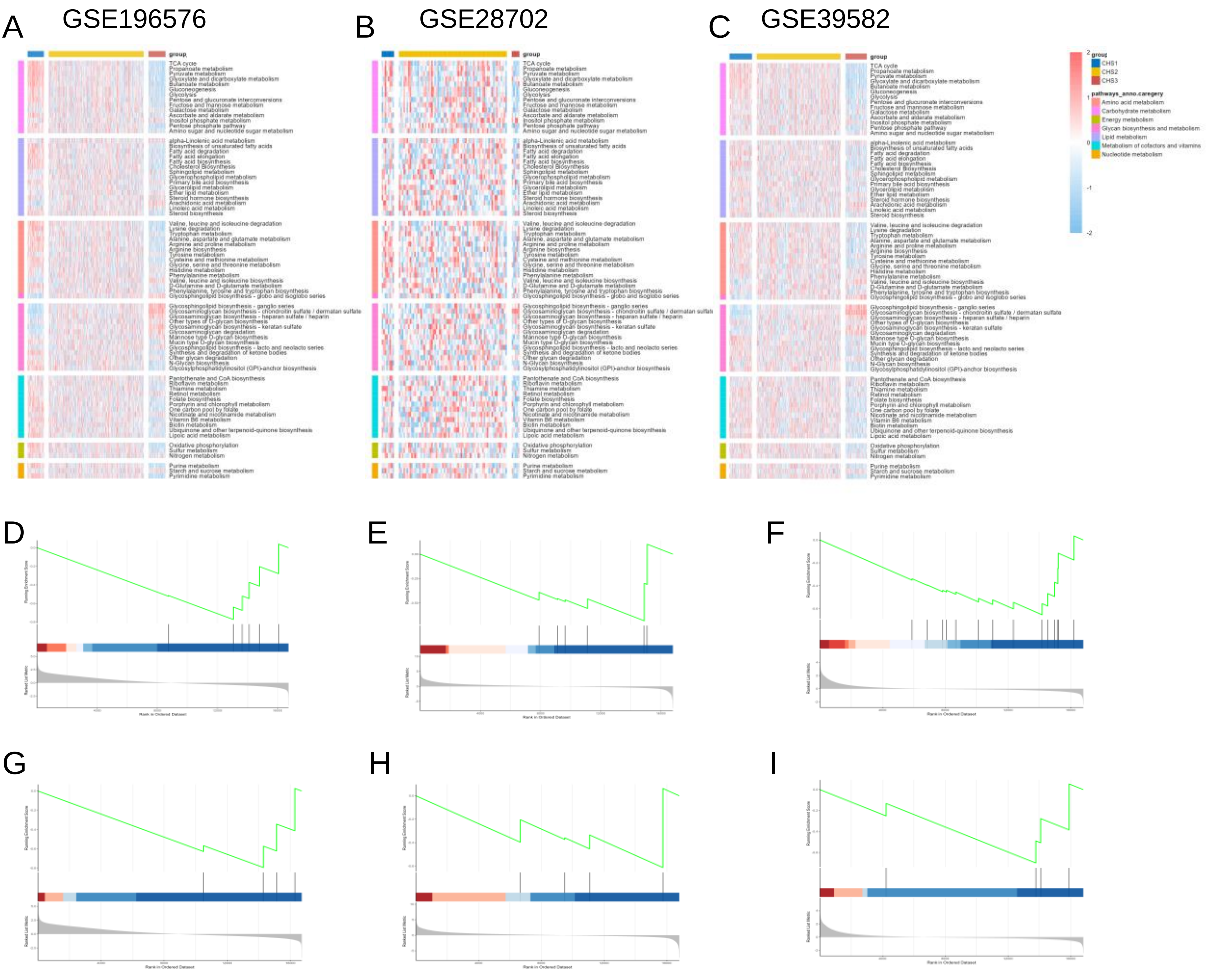
A
GSE196576
B
GSE28702
C
GSE39582
group
CHS1
CHS2
CHS3
pathways_anno.caregery
Amino acid metabolism
Carbohydrate metabolism
Energy metabolism
Glycan biosynthesis and metabolism
Lipid metabolism
Metabolism of cofactors and vitamins
Nucleotide metabolism
D
E
F
G
H
I

Figure S5

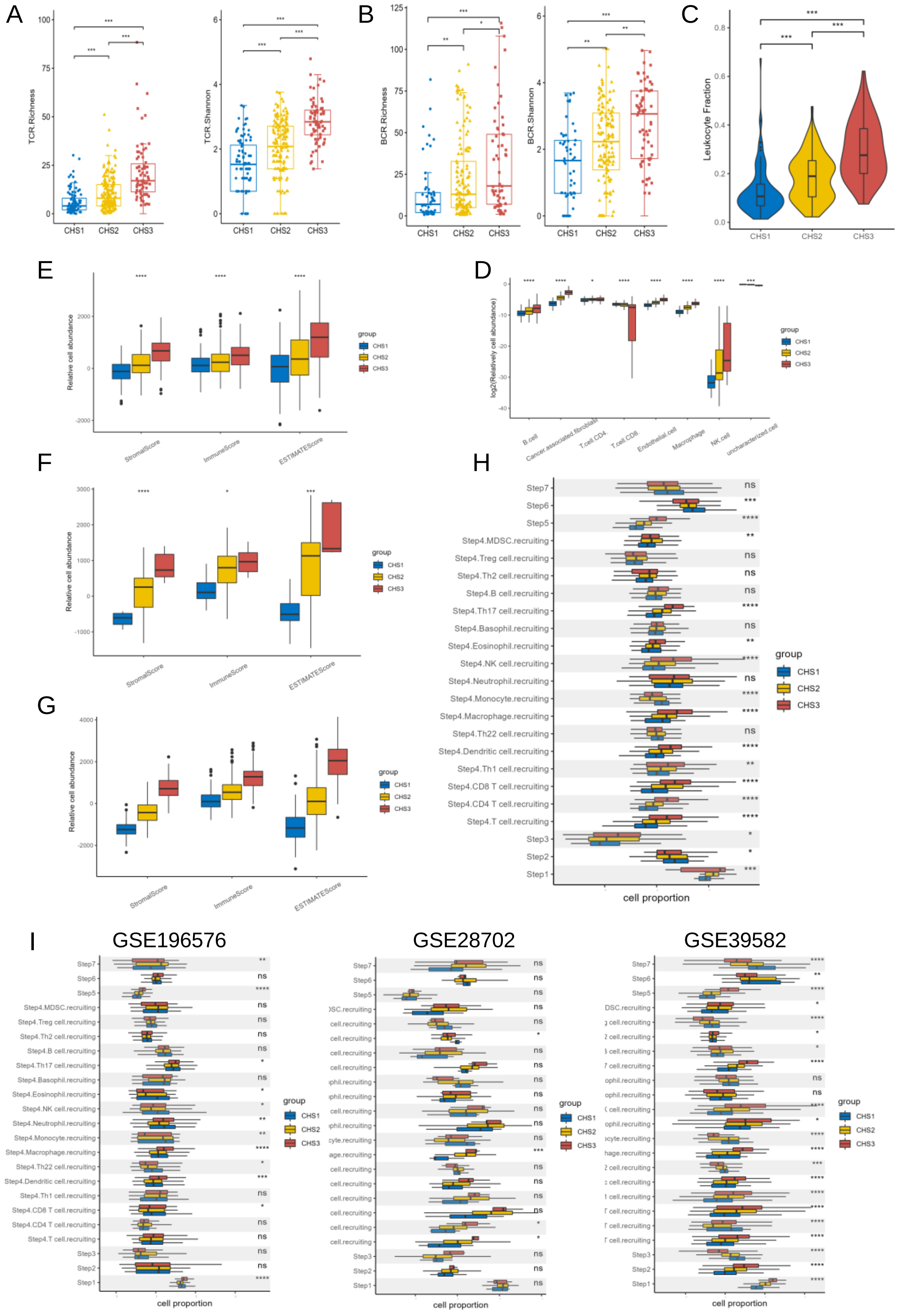
A
TCR.Richness
TCR.Shannon
B
BCR.Richness
BCR.Shannon
C
Leukocyte Fraction
CHS1
CHS2
CHS3
D
log2(Relatively cell abundance)
B.cell
Cancer.associated.fibroblast
T.cell.CD4
T.cell.CD8
Endothelial.cell
Macrophage
NK.cell
uncharacterized.cell
E
F
G
Relative cell abundance
StromalScore
ImmuneScore
ESTIMATEScore
group
H
Step7
Step6
Step5
Step4.MDSC.recruiting
Step4.Treg cell.recruiting
Step4.Th2 cell.recruiting
Step4.B cell.recruiting
Step4.Th17 cell.recruiting
Step4.Basophil.recruiting
Step4.Eosinophil.recruiting
Step4.NK cell.recruiting
Step4.Neutrophil.recruiting
Step4.Monocyte.recruiting
Step4.Macrophage.recruiting
Step4.Th22 cell.recruiting
Step4.Dendritic cell.recruiting
Step4.Th1 cell.recruiting
Step4.CD8 T cell.recruiting
Step4.CD4 T cell.recruiting
Step4.T cell.recruiting
Step3
Step2
Step1
cell proportion
I
GSE196576
GSE28702
GSE39582

Figure S6

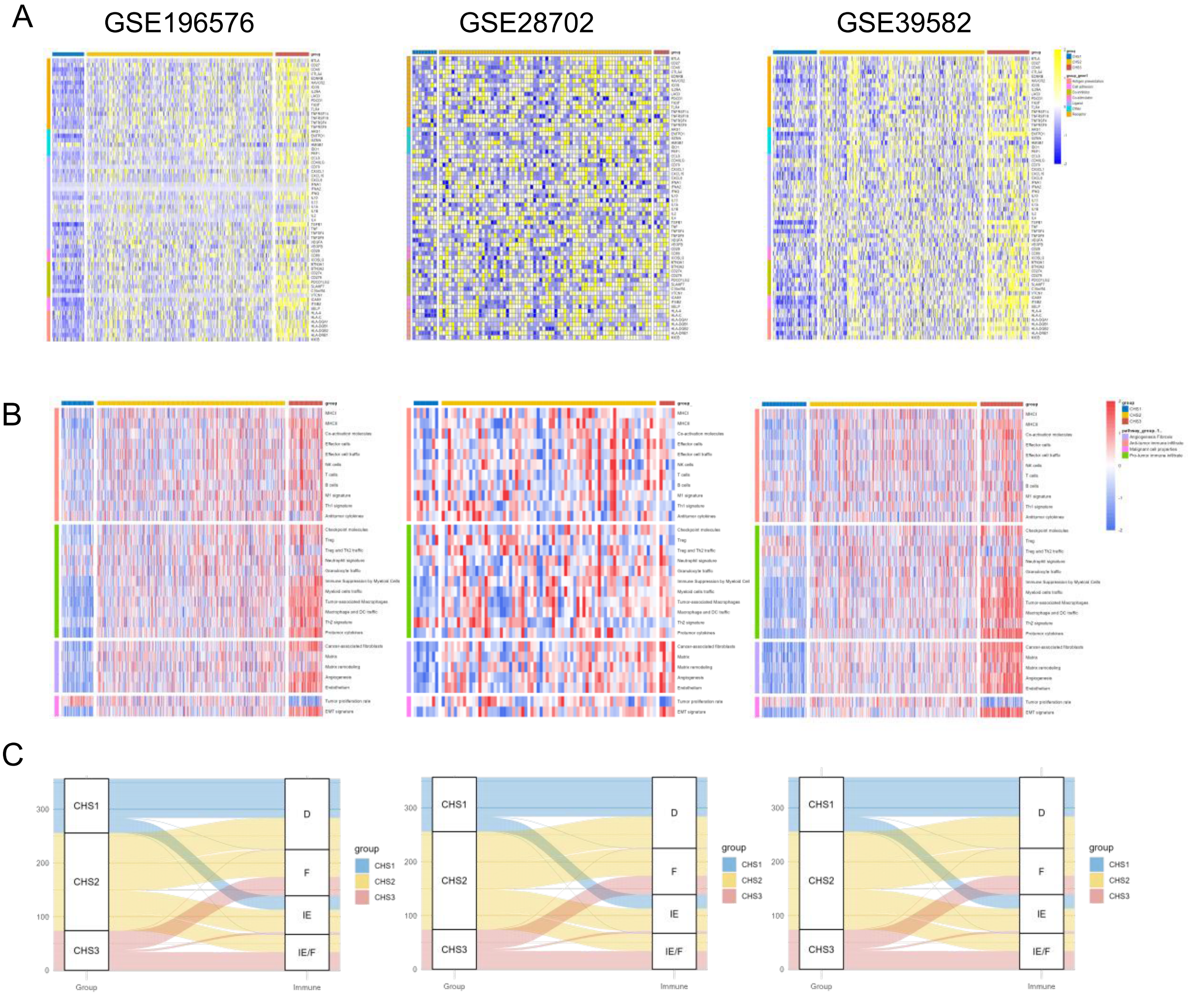
A
GSE196576
GSE28702
GSE39582
B
C
CHS1
CHS2
CHS3
D
F
IE
IE/F
group
CHS1
CHS2
CHS3
300
200
100
0
Group
Immune

Figure S7

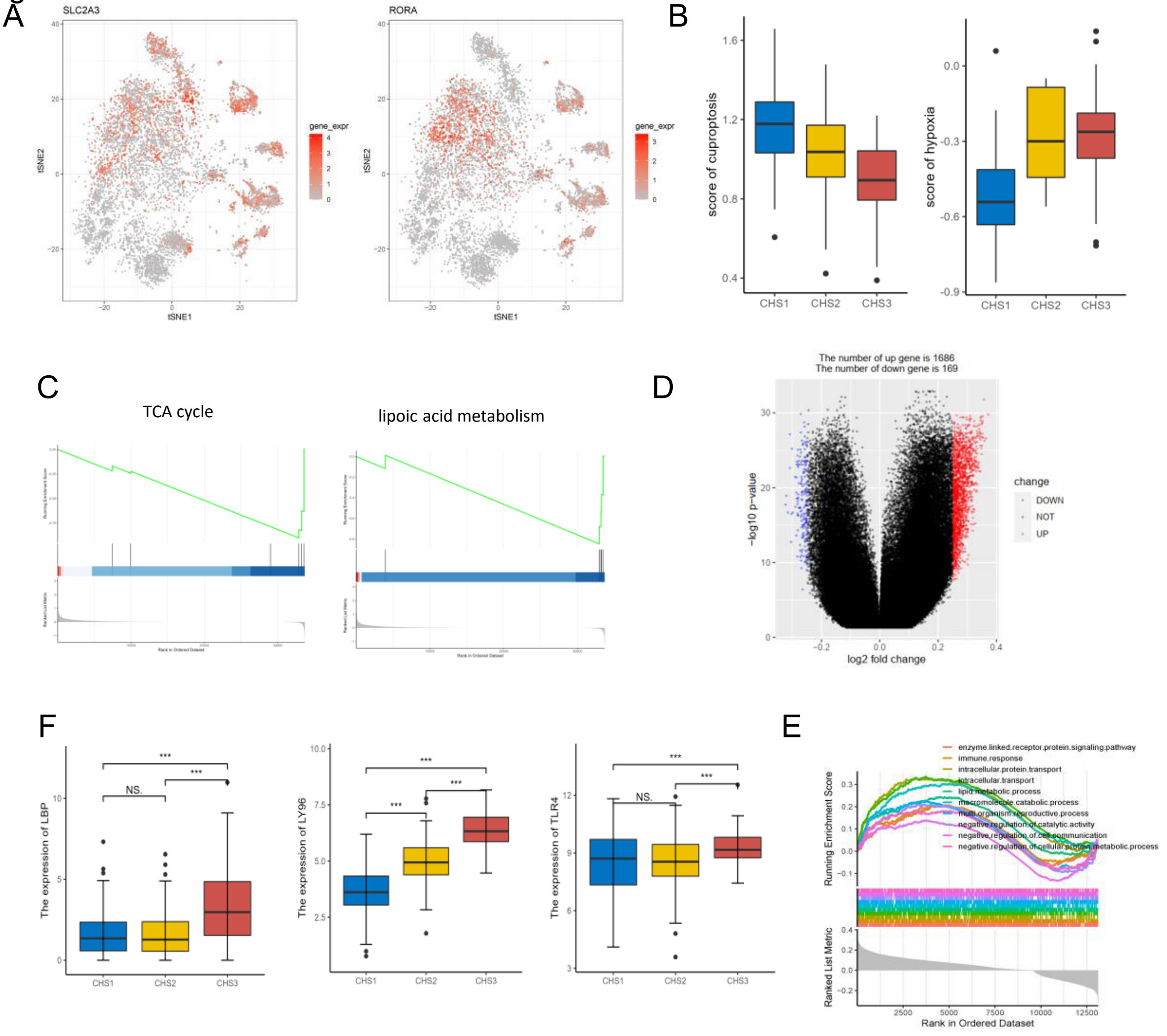
A
SLC2A3
RORA
tSNE1
tSNE2
gene_expr
B
score of cuproptosis
score of hypoxia
CHS1
CHS2
CHS3
C
TCA cycle
lipoic acid metabolism
Rank in Ordered Dataset
D
The number of up gene is 1686
The number of down gene is 169
-log10 p-value
log2 fold change
change
DOWN
NOT
UP
F
The expression of LBP
The expression of LY96
The expression of TLR4
NS.
***
E
enzyme.linked.receptor.protein.signaling.pathway
immune.response
intracellular.protein.transport
intracellular.transport
lipid.metabolic.process
macromolecule.catabolic.process
multi.organism.reproductive.process
negative.regulation.of.catalytic.activity
negative.regulation.of.cell.communication
negative.regulation.of.cellular.protein.metabolic.process
Running Enrichment Score
Ranked List Metric
Rank in Ordered Dataset

Figure S8

A

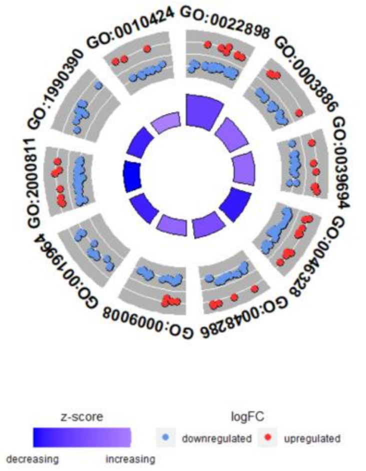

ID	Description
GO:0022898	regulation of transmembrane transporter activity
GO:0003886	DNA cytosine-5--methyltransferase activity
GO:0039694	viral RNA genome replication
GO:0046328	regulation of JNK cascade
GO:0048286	lung alveolus development
GO:0009008	DNA-methyltransferase activity
GO:0019964	interferon-gamma binding
GO:2000811	negative regulation of anoikis
GO:1990390	protein K33-linked ubiquitination
GO:0010424	DNA methylation on cytosine within a CG sequence

B

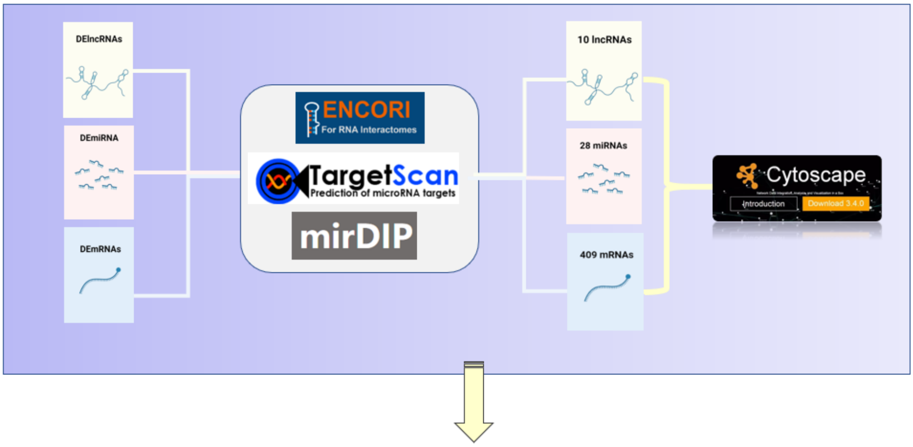

C

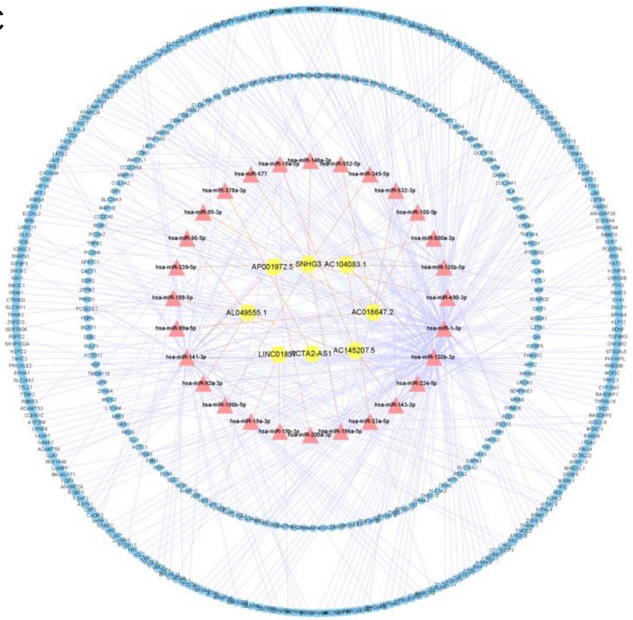

Figure S9

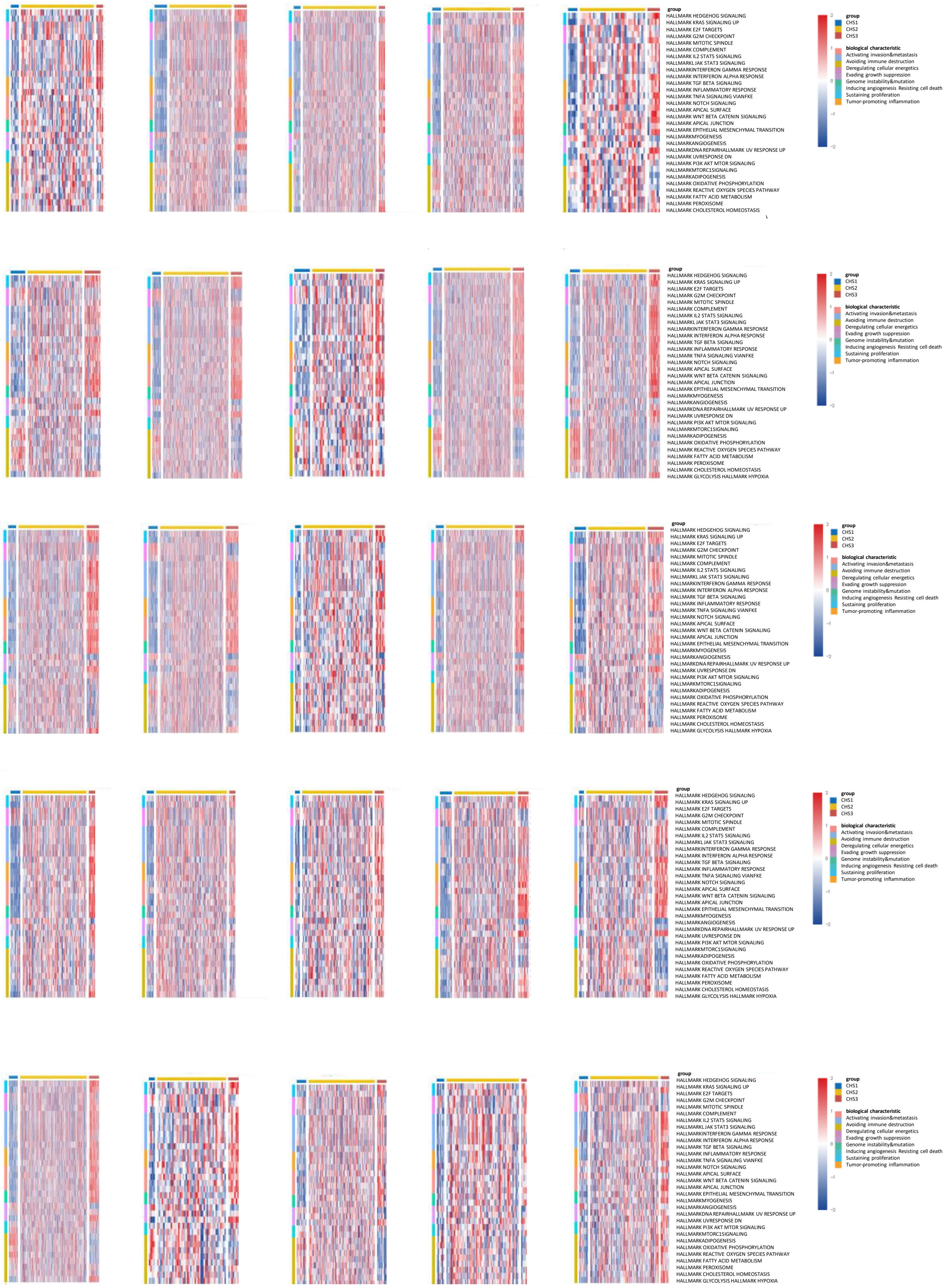
group
HALLMARK HEDGEHOG SIGNALING
HALLMARK KRAS SIGNALING UP
HALLMARK E2F TARGETS
HALLMARK G2M CHECKPOINT
HALLMARK MITOTIC SPINDLE
HALLMARK COMPLEMENT
HALLMARK IL2 STAT5 SIGNALING
HALLMARKL JAK STAT3 SIGNALING
HALLMARKINTERFERON GAMMA RESPONSE
HALLMARK INTERFERON ALPHA RESPONSE
HALLMARK TGF BETA SIGNALING
HALLMARK INFLAMMATORY RESPONSE
HALLMARK TNFA SIGNALING VIANFKE
HALLMARK NOTCH SIGNALING
HALLMARK APICAL SURFACE
HALLMARK WNT BETA CATENIN SIGNALING
HALLMARK APICAL JUNCTION
HALLMARK EPITHELIAL MESENCHYMAL TRANSITION
HALLMARKMYOGENESIS
HALLMARKANGIOGENESIS
HALLMARKDNA REPAIRHALLMARK UV RESPONSE UP
HALLMARK UVRESPONSE DN
HALLMARK PI3K AKT MTOR SIGNALING
HALLMARKMTORC1SIGNALING
HALLMARKADIPOGENESIS
HALLMARK OXIDATIVE PHOSPHORYLATION
HALLMARK REACTIVE OXYGEN SPECIES PATHWAY
HALLMARK FATTY ACID METABOLISM
HALLMARK PEROXISOME
HALLMARK CHOLESTEROL HOMEOSTASIS
HALLMARK GLYCOLYSIS HALLMARK HYPOXIA
2
1
0
-1
-2
group
CHS1
CHS2
CHS3
biological characteristic
Activating invasion&metastasis
Avoiding immune destruction
Deregulating cellular energetics
Evading growth suppression
Genome instability&mutation
Inducing angiogenesis Resisting cell death
Sustaining proliferation
Tumor-promoting inflammation

Figure S10

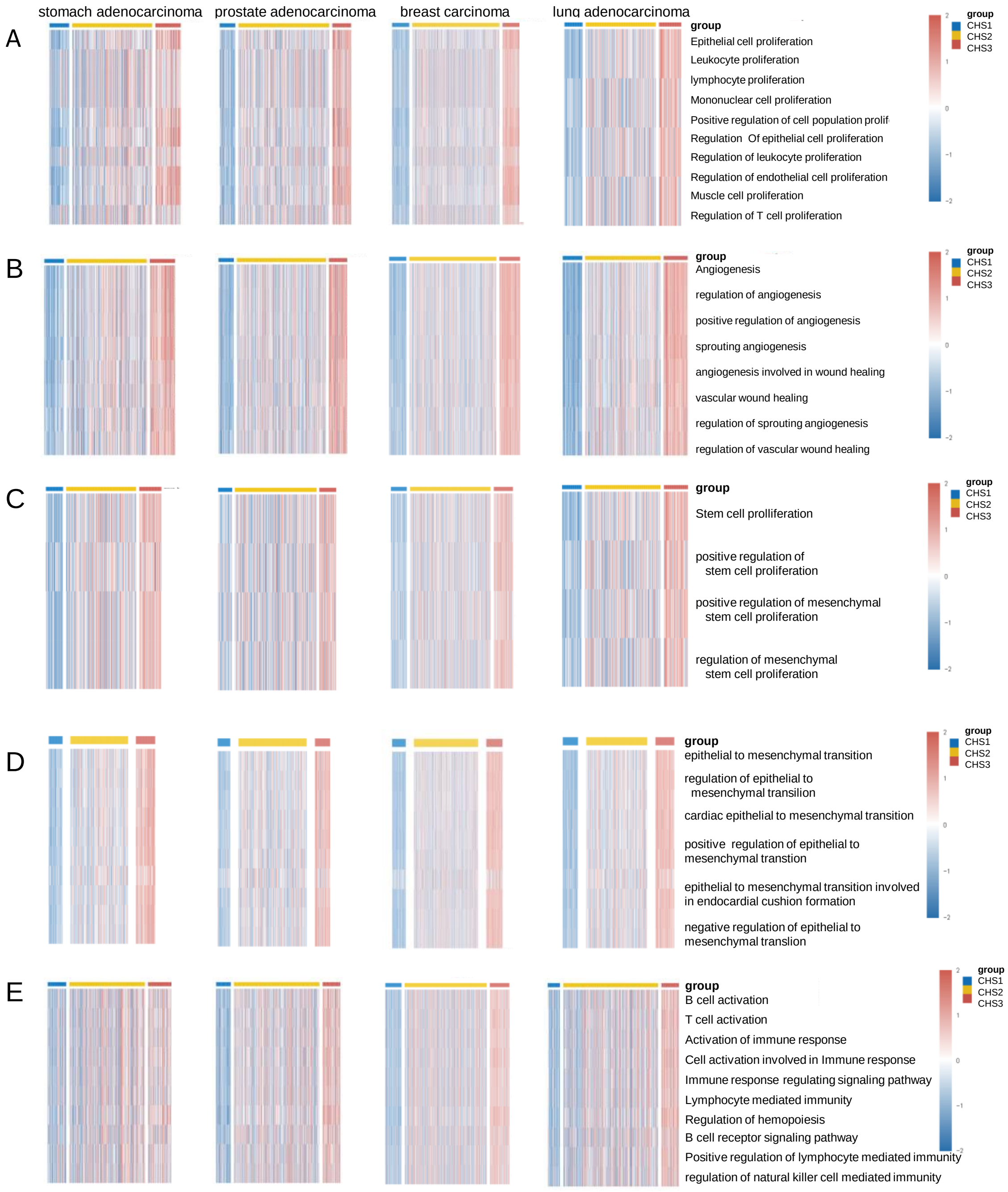

stomach adenocarcinoma
prostate adenocarcinoma
breast carcinoma
lung adenocarcinoma
A
group
Epithelial cell proliferation
Leukocyte proliferation
lymphocyte proliferation
Mononuclear cell proliferation
Positive regulation of cell population prolif
Regulation Of epithelial cell proliferation
Regulation of leukocyte proliferation
Regulation of endothelial cell proliferation
Muscle cell proliferation
Regulation of T cell proliferation
group
CHS1
CHS2
CHS3
B
group
Angiogenesis
regulation of angiogenesis
positive regulation of angiogenesis
sprouting angiogenesis
angiogenesis involved in wound healing
vascular wound healing
regulation of sprouting angiogenesis
regulation of vascular wound healing
C
group
Stem cell prolliferation
positive regulation of stem cell proliferation
positive regulation of mesenchymal stem cell proliferation
regulation of mesenchymal stem cell proliferation
D
group
epithelial to mesenchymal transition
regulation of epithelial to mesenchymal transilion
cardiac epithelial to mesenchymal transition
positive regulation of epithelial to mesenchymal transtion
epithelial to mesenchymal transition involved in endocardial cushion formation
negative regulation of epithelial to mesenchymal translion
E
group
B cell activation
T cell activation
Activation of immune response
Cell activation involved in Immune response
Immune response regulating signaling pathway
Lymphocyte mediated immunity
Regulation of hemopoiesis
B cell receptor signaling pathway
Positive regulation of lymphocyte mediated immunity
regulation of natural killer cell mediated immunity

Figure S11

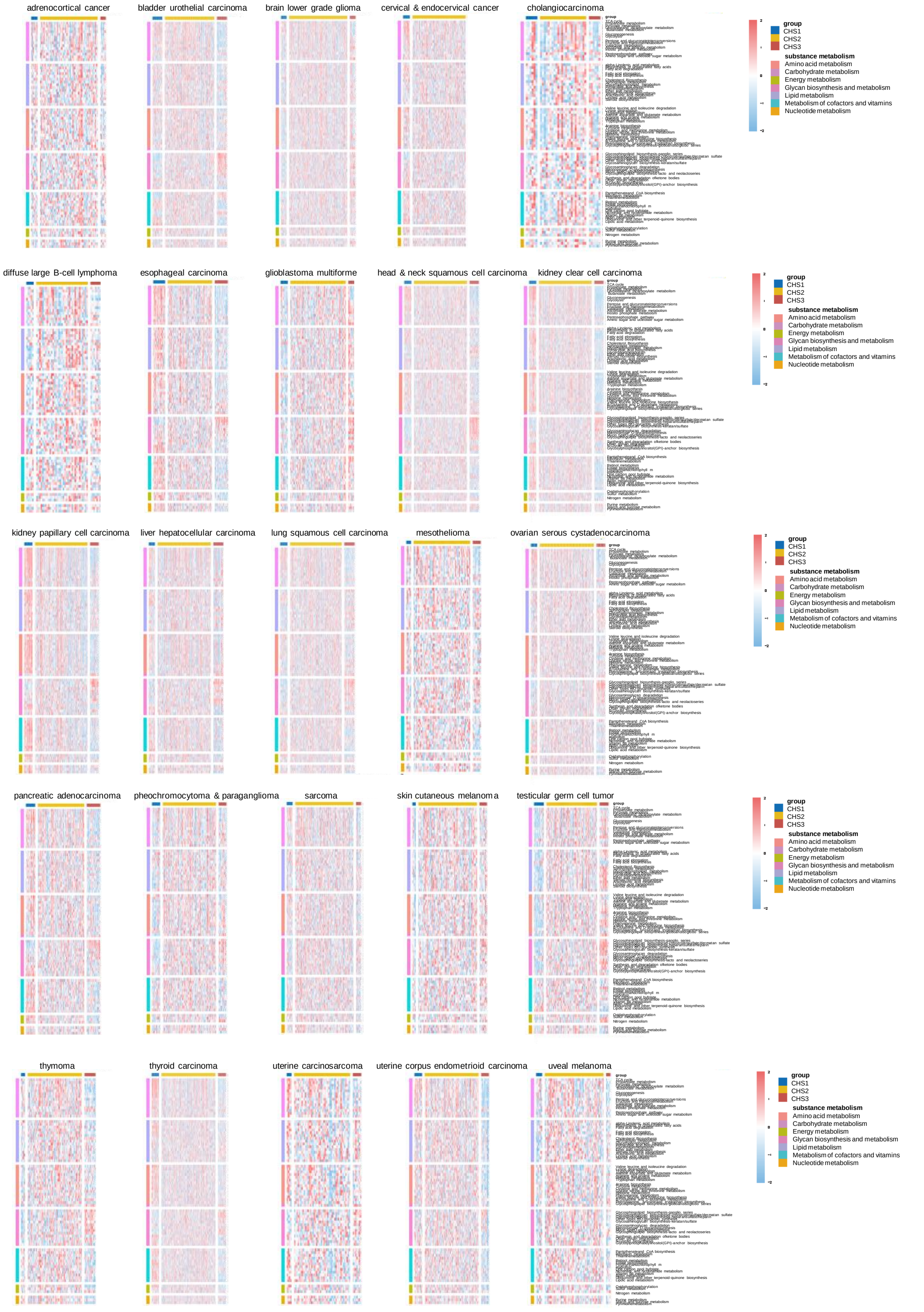
adrenocortical cancer
bladder urothelial carcinoma
brain lower grade glioma
cervical & endocervical cancer
cholangiocarcinoma
diffuse large B-cell lymphoma
esophageal carcinoma
glioblastoma multiforme
head & neck squamous cell carcinoma
kidney clear cell carcinoma
kidney papillary cell carcinoma
liver hepatocellular carcinoma
lung squamous cell carcinoma
mesothelioma
ovarian serous cystadenocarcinoma
pancreatic adenocarcinoma
pheochromocytoma & paraganglioma
sarcoma
skin cutaneous melanoma
testicular germ cell tumor
thymoma
thyroid carcinoma
uterine carcinosarcoma
uterine corpus endometrioid carcinoma
uveal melanoma
group
CHS1
CHS2
CHS3
substance metabolism
Amino acid metabolism
Carbohydrate metabolism
Energy metabolism
Glycan biosynthesis and metabolism
Lipid metabolism
Metabolism of cofactors and vitamins
Nucleotide metabolism

Figure S12

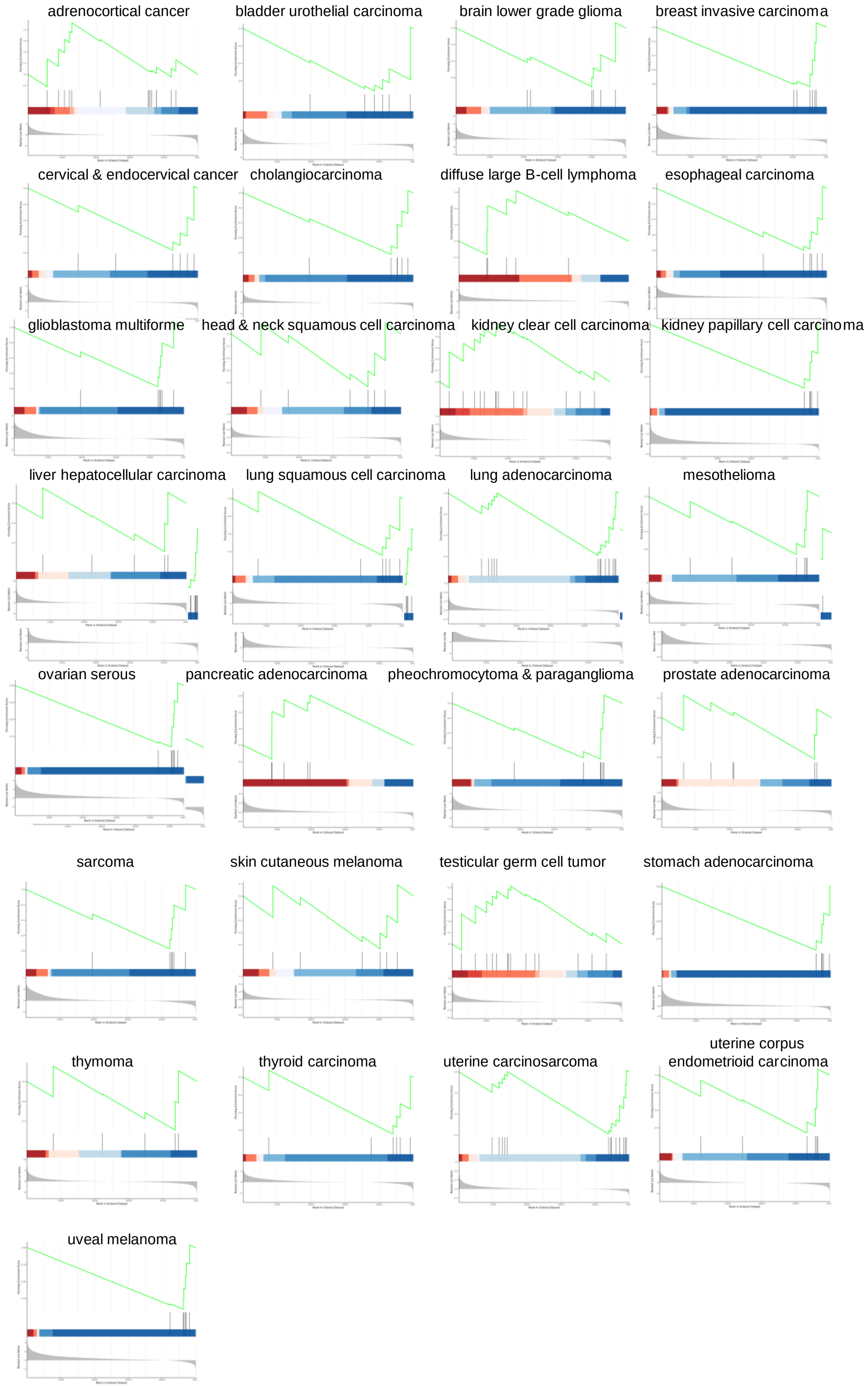
adrenocortical cancer
bladder urothelial carcinoma
brain lower grade glioma
breast invasive carcinoma
cervical & endocervical cancer
cholangiocarcinoma
diffuse large B-cell lymphoma
esophageal carcinoma
glioblastoma multiforme
head & neck squamous cell carcinoma
kidney clear cell carcinoma
kidney papillary cell carcinoma
liver hepatocellular carcinoma
lung squamous cell carcinoma
lung adenocarcinoma
mesothelioma
ovarian serous
pancreatic adenocarcinoma
pheochromocytoma & paraganglioma
prostate adenocarcinoma
sarcoma
skin cutaneous melanoma
testicular germ cell tumor
stomach adenocarcinoma
thymoma
thyroid carcinoma
uterine carcinosarcoma
uterine corpus endometrioid carcinoma
uveal melanoma

Figure S13

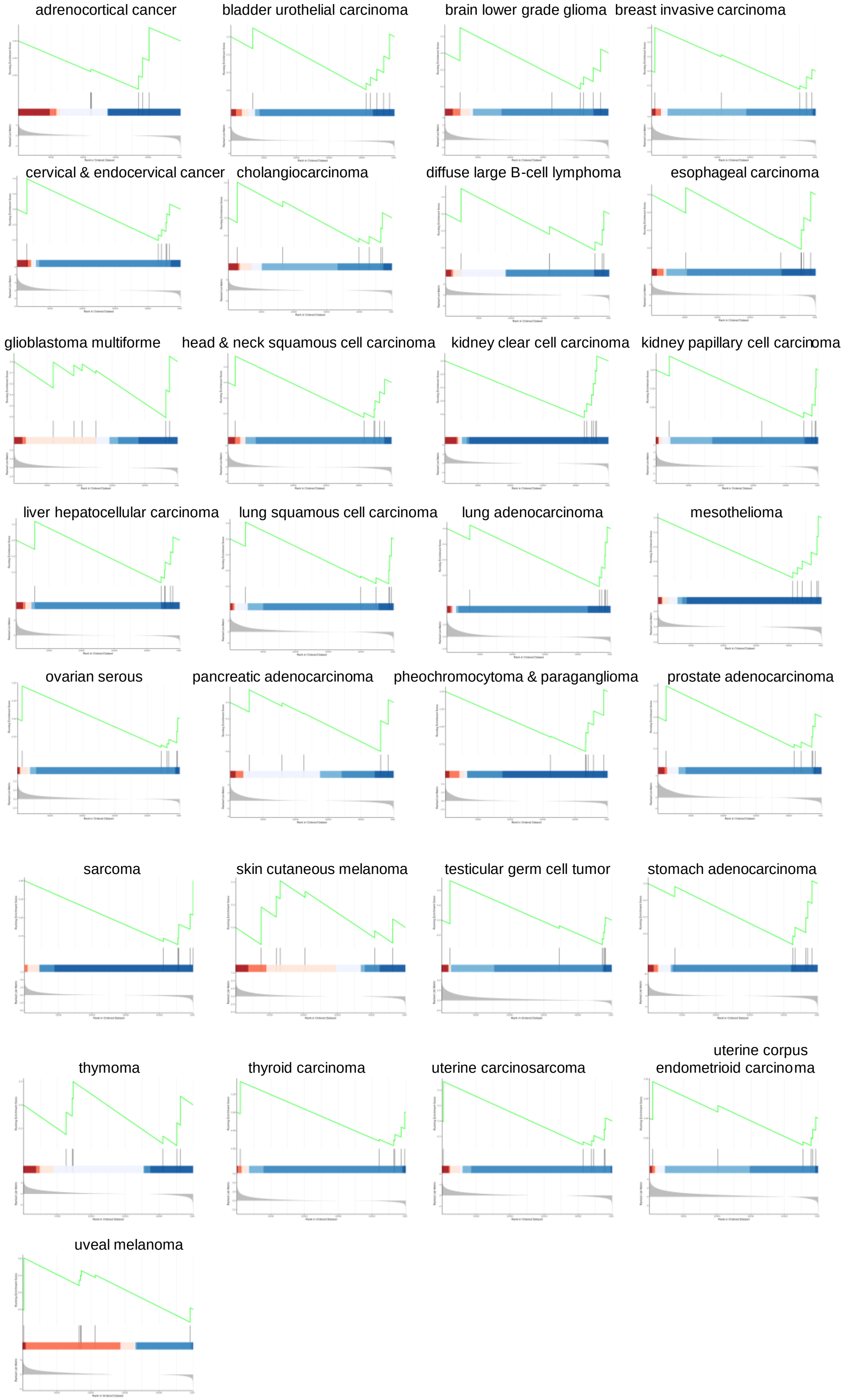
adrenocortical cancer
bladder urothelial carcinoma
brain lower grade glioma
breast invasive carcinoma
cervical & endocervical cancer
cholangiocarcinoma
diffuse large B-cell lymphoma
esophageal carcinoma
glioblastoma multiforme
head & neck squamous cell carcinoma
kidney clear cell carcinoma
kidney papillary cell carcinoma
liver hepatocellular carcinoma
lung squamous cell carcinoma
lung adenocarcinoma
mesothelioma
ovarian serous
pancreatic adenocarcinoma
pheochromocytoma & paraganglioma
prostate adenocarcinoma
sarcoma
skin cutaneous melanoma
testicular germ cell tumor
stomach adenocarcinoma
thymoma
thyroid carcinoma
uterine carcinosarcoma
uterine corpus endometrioid carcinoma
uveal melanoma

Figure S14

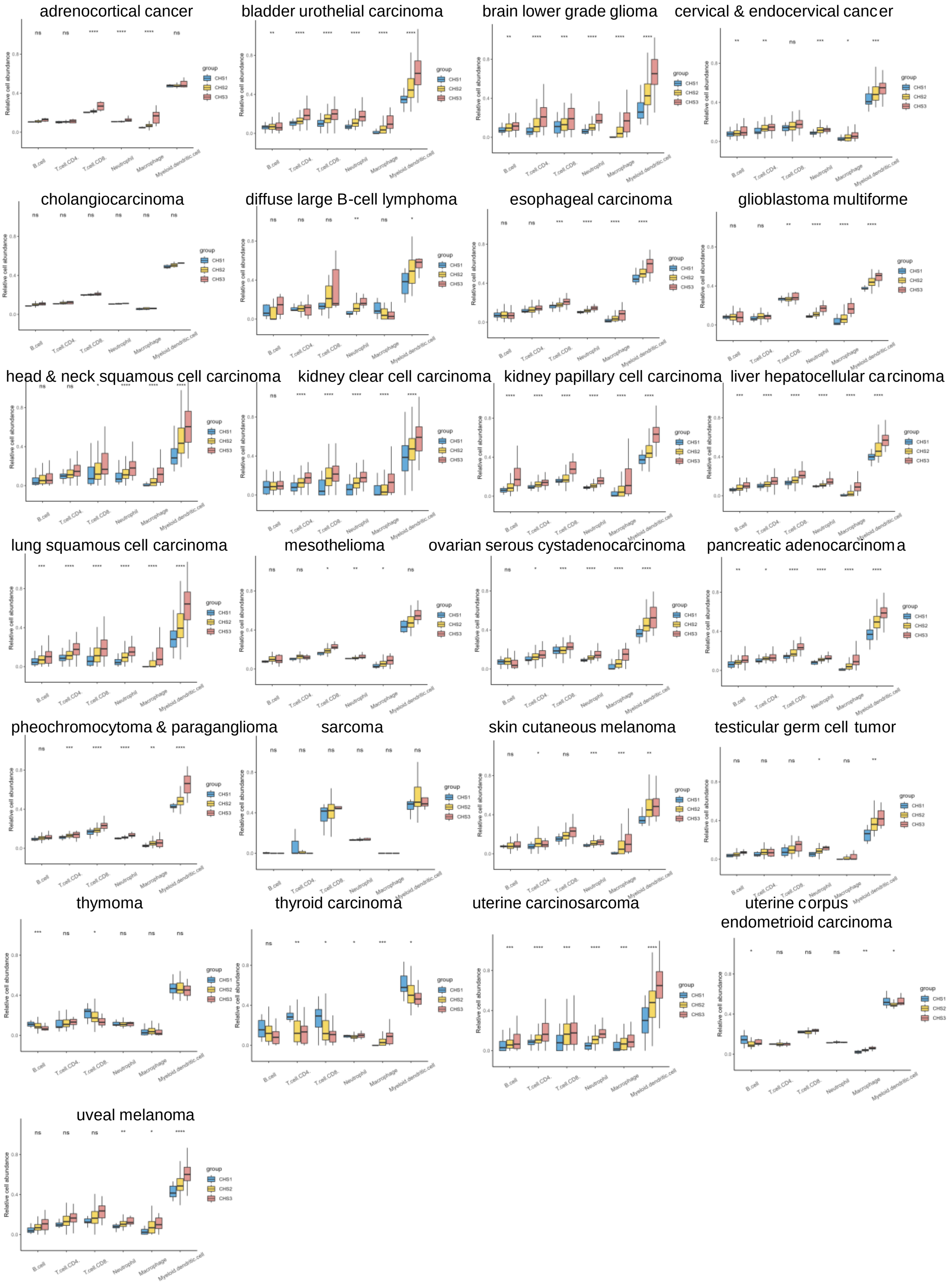
adrenocortical cancer
bladder urothelial carcinoma
brain lower grade glioma
cervical & endocervical cancer
cholangiocarcinoma
diffuse large B-cell lymphoma
esophageal carcinoma
glioblastoma multiforme
head & neck squamous cell carcinoma
kidney clear cell carcinoma
kidney papillary cell carcinoma
liver hepatocellular carcinoma
lung squamous cell carcinoma
mesothelioma
ovarian serous cystadenocarcinoma
pancreatic adenocarcinoma
pheochromocytoma & paraganglioma
sarcoma
skin cutaneous melanoma
testicular germ cell tumor
thymoma
thyroid carcinoma
uterine carcinosarcoma
uterine corpus endometrioid carcinoma
uveal melanoma
Relative cell abundance
group
CHS1
CHS2
CHS3
B.cell
T.cell.CD4
T.cell.CD8
Neutrophil
Macrophage
Myeloid.dendritic.cell

Figure S15

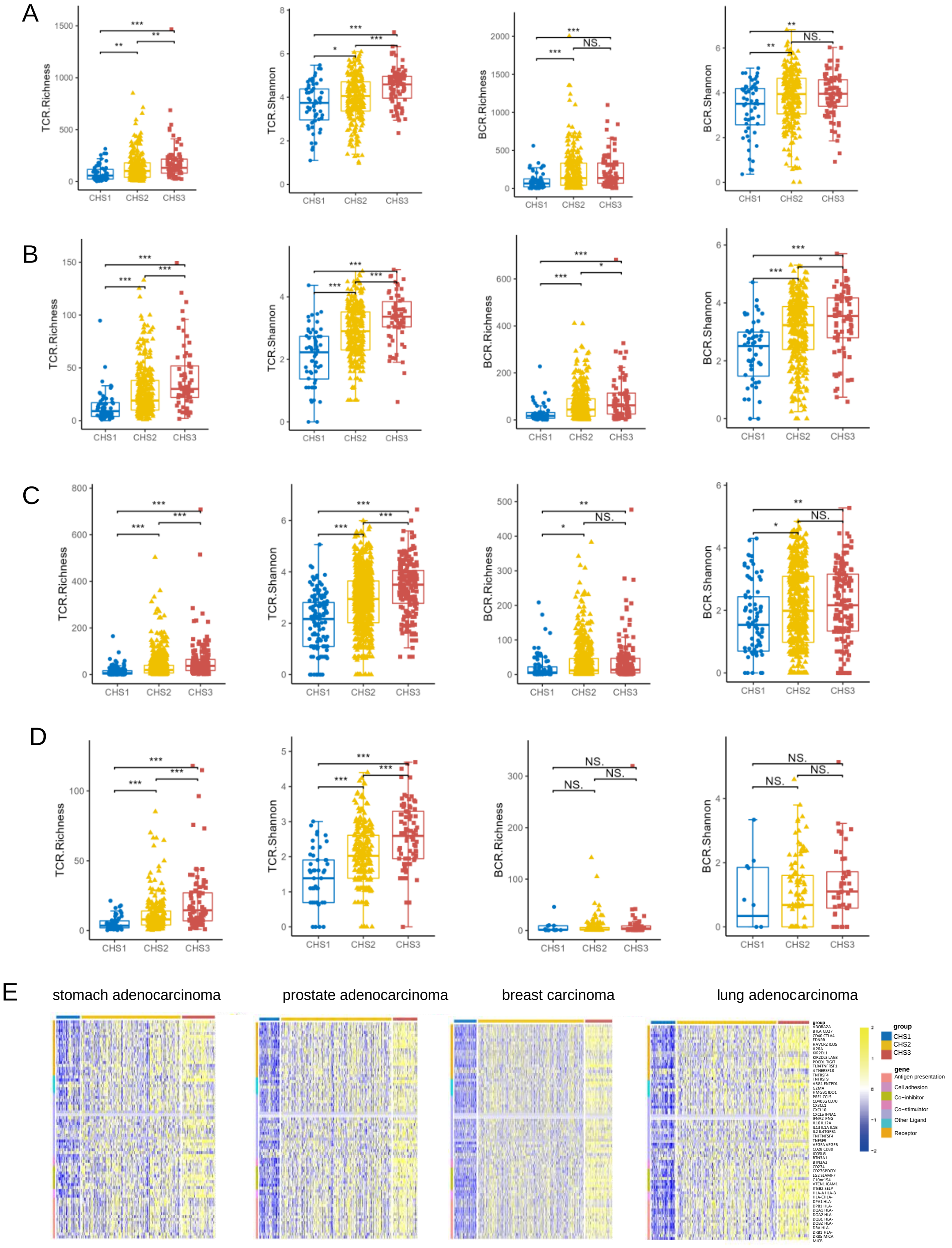
A
B
C
D
TCR.Richness
TCR.Shannon
BCR.Richness
BCR.Shannon
CHS1
CHS2
CHS3
NS.
E
stomach adenocarcinoma
prostate adenocarcinoma
breast carcinoma
lung adenocarcinoma
group
gene
Antigen presentation
Cell adhesion
Co−inhibitor
Co−stimulator
Other Ligand
Receptor

Figure S16

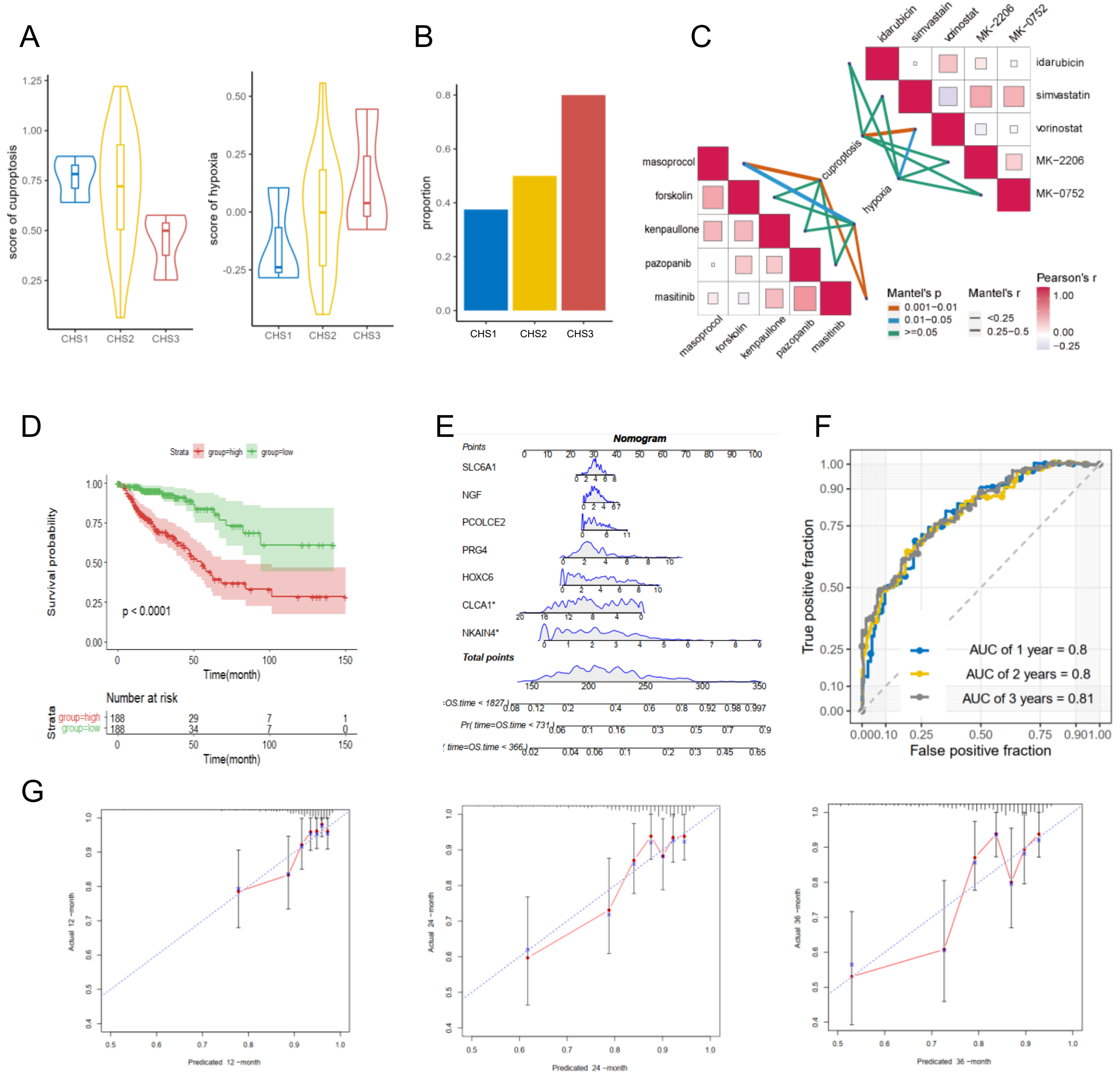
A
B
C
D
E
F
G
score of cuproptosis
score of hypoxia
CHS1
CHS2
CHS3
proportion
idarubicin
simvastatin
vorinostat
MK−2206
MK−0752
masoprocol
forskolin
kenpaullone
pazopanib
masitinib
cuproptosis
hypoxia
Mantel's p
0.001−0.01
0.01−0.05
>=0.05
Mantel's r
<0.25
0.25−0.5
Pearson's r
Strata
group=high
group=low
Survival probability
p < 0.0001
Time(month)
Number at risk
Nomogram
Points
SLC6A1
NGF
PCOLCE2
PRG4
HOXC6
CLCA1*
NKAIN4*
Total points
True positive fraction
False positive fraction
AUC of 1 year = 0.8
AUC of 2 years = 0.8
AUC of 3 years = 0.81
Actual 12 −month
Predicated 12 −month
Actual 24 −month
Predicated 24 −month
Actual 36 −month
Predicated 36 −month

Figure S17

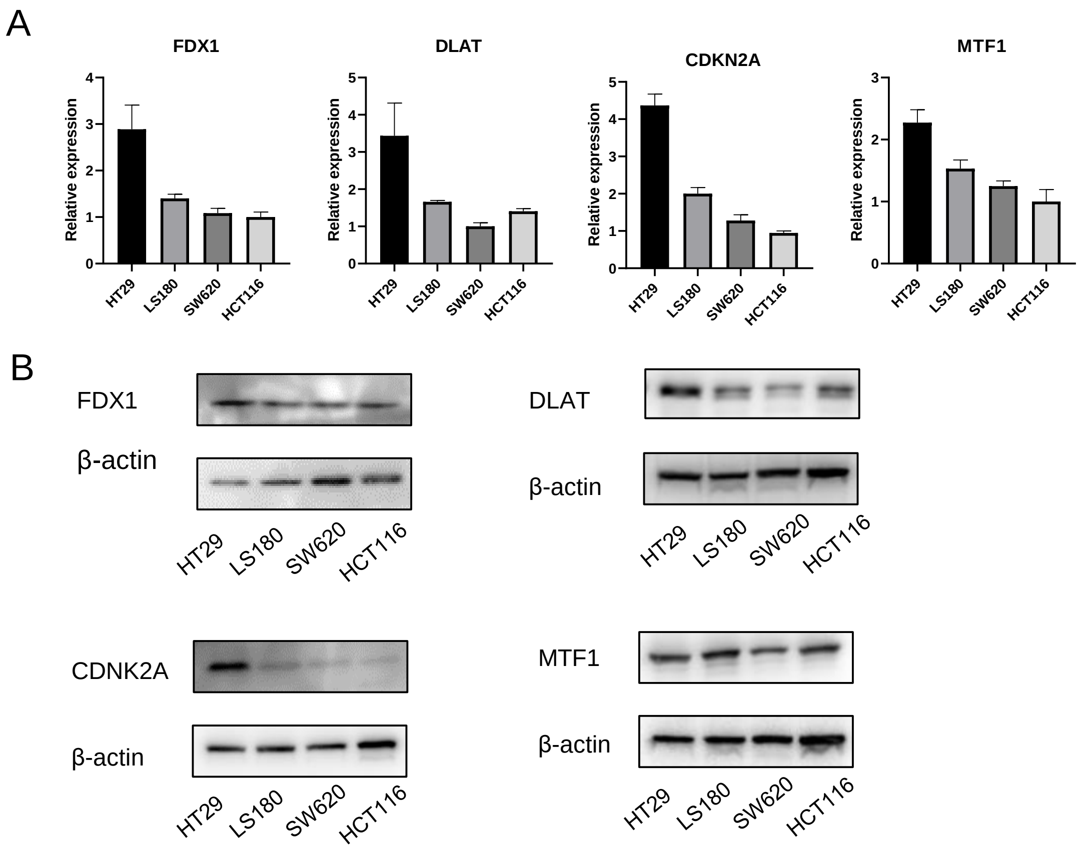
A
FDX1
DLAT
CDKN2A
MTF1
Relative expression
HT29
LS180
SW620
HCT116
B
FDX1
β-actin
DLAT
β-actin
CDNK2A
β-actin
MTF1
β-actin
HT29
LS180
SW620
HCT116

Figure S18

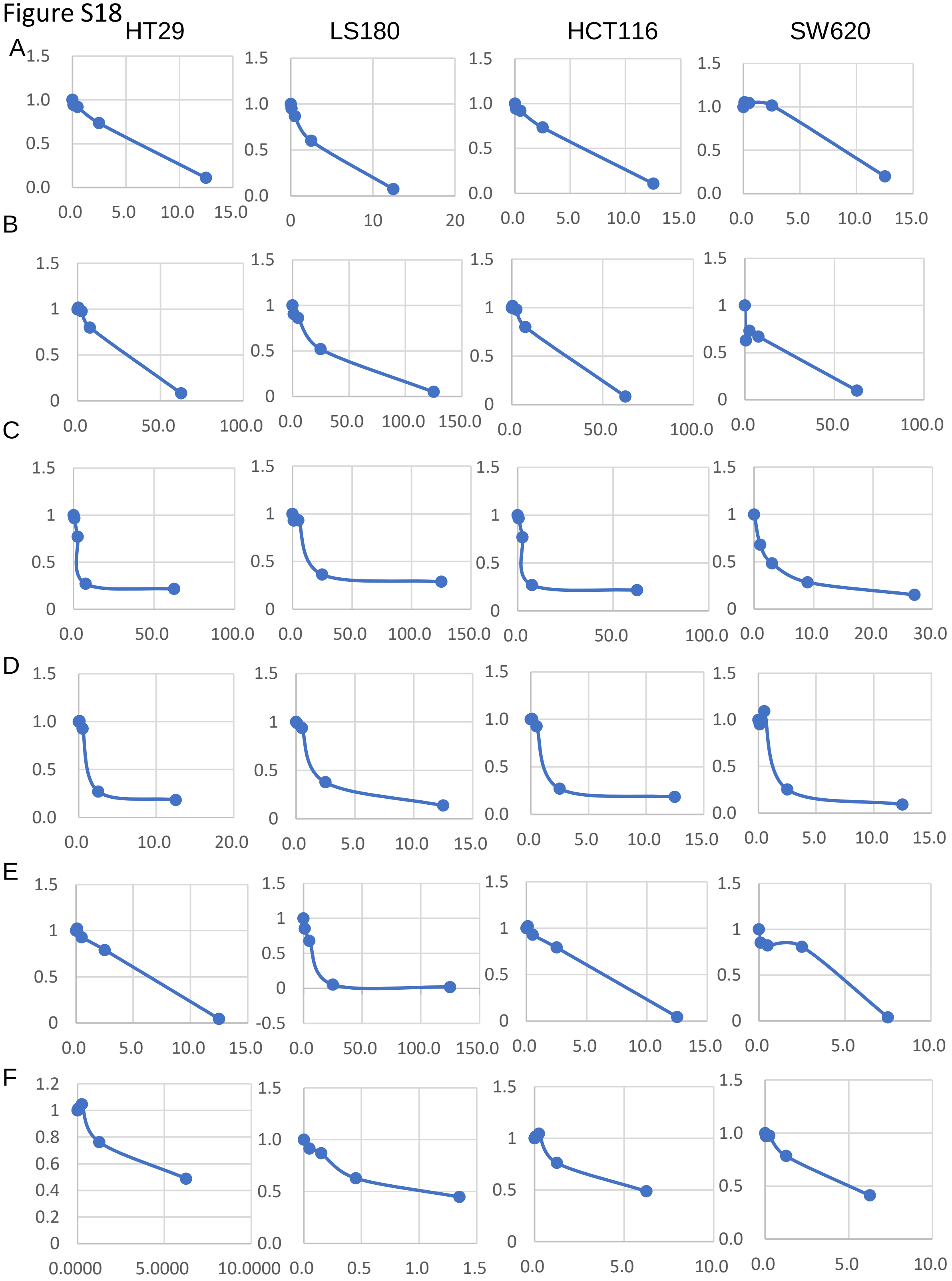

Table S1. The key genes of cuproptosis and hypoxia.

gene
FDX1
LIAS
LIPT1
DLD
DLAT
PDHA1
PDHB
SLC31A1
MTF1
GLS
CDKN2A
ATP7A
ATP7B
ADM
ADORA2B
AK3L1
AKAP12
ALDOA
ALDOB
ALDOC
AMPD3
ANGPTL4
ANKZF1
ANXA2
ATF3
ATP7A
B3GALT6
B4GALNT2
BCAN
BCL2
BGN
BHLHE40
BNIP3L
BRS3
BTG1
CA12
CASP6
CAV1
CCNG2
CCRN4L
CDKN1A
CDKN1B
CDKN1C
CHST2
CHST3
CITED2
COL5A1
CP
CSRP2
CTGF
CXCR4

CXCR7
CYR61
DCN
DDIT3
DDIT4
DPYSL4
DTNA
DUSP1
EDN2
EFNA1
EFNA3
EGFR
ENO1
ENO2
ENO3
ERO1L
ERRFI1
ETS1
EXT1
F3
FAM162A
FBP1
FOS
FOSL2
FOXO3
GAA
GALK1
GAPDH
GAPDHS
GBE1
GCK
GCNT2
GLRX
GPC1
GPC3
GPC4
GPI
GRHPR
GYS1
HAS1
HDLBP
HEXA
HK1
HK2
HMOX1
HOXB9
HS3ST1
HSPA5
IDS
IER3
IGFBP1
IGFBP3
IL6

ILVBL
INHA
IRS2
ISG20
JMJD6
JUN
KDELR3
KDM3A
KIF5A
KLF6
KLF7
KLHL24
LALBA
LARGE
LDHA
LDHC
LOX
LXN
MAFF
MAP3K1
MIF
MT1E
MT2A
MXI1
MYH9
NAGK
NCAN
NDRG1
NDST1
NDST2
NEDD4L
NFIL3
NR3C1
P4HA1
P4HA2
PAM
PCK1
PDGFB
PFKFB3
PFKL
PFKP
PGAM2
PGF
PGK1
PGM1
PGM2
PHKG1
PIM1
PKLR
PKP1
PLAC8
PLAUR
PLIN2

PNRC1
PPARGC1A
PPFIA4
PPP1R15A
PPP1R3C
PRDX5
PRKCA
PRKCDBP
PTRF
PYGM
RBPJ
RORA
RRAGD
S100A4
SAP30
SCARB1
SDC2
SDC3
SDC4
SELENBP1
SERPINE1
SIAH2
SLC25A1
SLC2A1
SLC2A3
SLC2A5
SLC37A4
SLC6A6
SRPX
STBD1
STC1
STC2
SULT2B1
TES
TGFB3
TGFBI
TGM2
TIPARP
TKTL1
TMEM45A
TNFAIP3
TPBG
TPD52
TPI1
TPST2
UGP2
VEGFA
VHL
VLDLR
WISP2
WSB1
XPNPEP1
ZFP36

ZNF292

Table S2. Primer sequences for qPCR

gene	Primer sequence (5’ ——3’)
FDX1	F:CCACTTTATAAACCGTGATGGTG
	R: ACATGCACCAAAGCCATCAA
DLAT	F: CGGAACTCCACGAGTGACC
	R: CCCCGCCATACCCTGTAGT
CDKN2A	F: CGCAGGTTCTTGGTCACTGT
	R: TGTTCACGAAAGCCAGAGCG
MTF1	F:CACAGTCCAGACAACAACATCA
	R: GCACCAGTCCGTTTTTATCCAC
GADPH	F: GGAGCGAGATCCCTCCAAAAT
	R: GGCTGTTGTCATACTTCTCATGG

Table S2. The lncRNA, miRNA and mRNA of the ceRNA network.

lnRNA	miRNA	mRNA
AC145207.5	hsa-miR-1-3p	TGFBR3
AC018647.2	hsa-miR-1-3p	ARHGAP29
AC104083.1	hsa-miR-1-3p	ZNF281
SNHG3	hsa-miR-1-3p	ETNK2
AP001972.5	hsa-miR-1-3p	PRDM16
AL049555.1	hsa-miR-1-3p	CLIC4
LINC01857	hsa-miR-1-3p	PRKAA2
ACTA2-AS1	hsa-miR-1-3p	ROR1
	hsa-miR-1-3p	NAV1
	hsa-miR-130b-3p	ATP2B4
	hsa-miR-135b-5p	NFASC
	hsa-miR-141-3p	DIP2C
	hsa-miR-143-3p	NRP1
	hsa-miR-148a-3p	ZCCHC24
	hsa-miR-188-5p	ARID5B
	hsa-miR-18a-5p	DUSP5
	hsa-miR-196a-5p	BDNF
	hsa-miR-19a-3p	RAB30
	hsa-miR-224-5p	CADM1
	hsa-miR-339-5p	BACE1
	hsa-miR-33a-5p	ETS1
	hsa-miR-345-5p	TUB
	hsa-miR-378a-3p	AMOTL1
	hsa-miR-92a-3p	SOX5
		BCAT1
		CPNE8
		ITGA5
		NUAK1
		TSPAN9
		RASSF8
		NAV3
		MAB21L1
		KLF12
		NBEA
		CFL2
		ZFP36L1
		TTC7B
		NPAS3
		PELI2
		HIF1A
		SMOC1
		VASH1
		FBN1
		MYO5A
		RORA
		THBS1
		TMOD2
		AKAP13
		MAF
		ZBTB4
		RAB34

HS3ST3B1
MAP3K3
AXL
CCDC88A
AFF3
ZEB2
NR4A2
FIGN
COL5A2
HECW2
IKZF2
FN1
IGFBP5
EPHA4
LBH
MEIS1
INHBB
CYBRD1
NRP2
TGM2
RIMS4
INSM1
ADAMTS5
ERG
TIMP3
SH3BP5
THRB
TRANK1
DCBLD2
FSTL1
MYLK
RARB
STAC
PHLDB2
CLDN11
PDE5A
PCDH7
CTNND2
LIFR
IL6ST
EFNA5
MCC
SPOCK1
NR3C1
RAI14
VCAN
ATXN1
TRERF1
RRAGD
BACH2
SGK1
CITED2
RGS17

SLC35F1
GJA1
EYA4
PDE7B
TNFAIP3
UST
ESR1
QKI
GLI3
MAGI2
CDK14
FOXP2
DLC1
SNAI2
MMP16
TRPS1
SULF1
ZFHX4
SDC2
BNC2
ABCA1
NR4A3
ZNF618
CHD5
TNFSF4
CEP170
AKT3
S1PR1
PRRX1
TGFB2
FZD8
EGR2
SH3PXD2A
EBF3
ZEB1
MPPED2
CHST1
SESN3
RDX
TSPAN18
FAT3
NTM
CD69
BHLHE41
PRICKLE1
CPM
IGF1
ELK3
HCFC2
FGF9
FRY
SLAIN1
NOVA1

SIX4
FRMD6
RASGRP1
CPEB1
RGMA
RAB8B
FOXF1
MYH10
NPTX1
TAOK1
NOG
TANC2
ANKRD29
BCL2
DSEL
TUBB6
MAPK4
NFIC
CYP26B1
IRS1
SOX11
CLIP4
ARHGEF4
CSRNP3
MAFB
SLC24A3
IQSEC1
PRICKLE2
ROBO1
HEG1
CNTN4
ITPR1
MITF
MRAS
TRPC3
PDGFRA
WWC2
STOX2
HAND1
SNX18
RNF180
MAP1B
SNAP91
EPHA7
FOXF2
SOBP
ETV1
PODXL
CREB5
SERPINE1
MDFIC
SOX7
EGR3

PURG
RUNX1T1
GDF6
LRP12
HAS2
ZFPM2
SLC24A2
GAS1
VLDLR
TGFBR1
PBX3
WNK3
LRCH2
RAB39B
ASAP3
SPSB1
RGL1
DENND5B
ZNF385A
ANO4
PCDH9
KCTD12
MEIS2
SV2B
CDYL2
NLGN2
CHST10
RBMS1
ITGAV
GULP1
WWTR1
TRPC1
SCHIP1
MEF2C
EBF1
CAMK4
ELOVL2
TRAM2
RNF144B
AKAP12
FKBP14
DOCK4
FZD1
PEG10
LZTS1
EYA1
SLA
NEFM
PI15
PAPPA
TSC22D3
FRMPD4
MSN

TTLL7
ASTN1
PGBD5
MAP7D1
MACF1
RGS2
MARK1
PHYHIPL
RBM20
ADRB1
NRIP3
DKK3
NCAM1
LMO3
LATS2
PRKD1
TTBK2
CSF3
ZNF521
TSHZ3
PEG3
CDC42EP3
NRXN1
ST3GAL5
ANKRD44
DOCK10
PLCL1
MAP2
MN1
TTC28
CCDC80
PCOLCE2
RBMS3
CADM2
BEND4
GPM6A
SORBS2
LIMCH1
ANK2
SYNPO2
ST8SIA4
NPR3
LAMA4
KIAA0408
PDE10A
FOXC1
JAZF1
IGFBP3
HGF
RELN
BHLHE22
GPM6B
ARMCX2

SLC16A2
RAB3B
PTGER3
COL24A1
WASF3
TMEM121
COL1A1
ITGB3
DTNA
NFATC1
ZNF570
LSAMP
ZBTB47
BSN
PDZD2
SLC1A3
NIPAL4
MDGA1
RAB23
DAAM2
FAM167A
SLC31A2
DNAJB4
CDH2
TRIB2
SERTAD4
B4GALNT1
PCDH17
CBFA2T3
ARHGAP28
DOK6
ADD2
MGAT3
PTX3
TCF21
LRRC4
SLITRK4
CACNA1H
KLHL14
CACNB4
SHOX2
ADAMTS3
TLL1
PRDM6
HDAC9
PAX5
HOXC8
IGDCC4
LRRC4B
KLF2
COL3A1
EPHA3
GDNF

OSMR
COL1A2
LRRC17
RSPO2
L1CAM
SAMD4A
NTRK3
ZNF385B
RAI2
CHIT1
MPEG1
TRIM9
ZNF423
HOXD10
BCL6
ARL10
CHST7
PDPN
TMEM119
BRSK1
UNC5C
NKX3-1
FHL1
ALPK3
RASGRP3
KCNIP3
ROBO2
SSBP2
TMED7-TICAM2
DNM1
ELAVL4
DACT1
CDKN1C
ACTC1
CD226
ST6GAL2
CBLN4
GAP43
FAM20C
CNKSR2
hsa-miR-1-3p
hsa-miR-100-5p
hsa-miR-130b-3p
hsa-miR-135b-5p
hsa-miR-141-3p
hsa-miR-143-3p
hsa-miR-148a-3p
hsa-miR-188-5p
hsa-miR-18a-5p
hsa-miR-196a-5p
hsa-miR-196b-5p
hsa-miR-19a-3p
hsa-miR-19b-3p

hsa-miR-200a-3p
hsa-miR-224-5p
hsa-miR-339-5p
hsa-miR-33a-5p
hsa-miR-345-5p
hsa-miR-378a-3p
hsa-miR-490-3p
hsa-miR-500a-3p
hsa-miR-532-3p
hsa-miR-552-5p
hsa-miR-577
hsa-miR-92a-3p
hsa-miR-95-3p
hsa-miR-96-5p
hsa-miR-99a-5p

Table S4. The category of CRC cell lines.

group	cell line
CHS1	SNU-283
CHS1	SNU-1040
CHS1	HT-29
CHS1	CL-40
CHS1	CW-2
CHS1	C125PM
CHS1	HT55
CHS1	ECC4
CHS1	NCI-H716
CHS1	NCI-H508
CHS1	SNU-C1
CHS1	SNU-61
CHS1	SNU-175
CHS1	HCC-56
CHS1	C99
CHS1	SK-CO-1
CHS1	T84
CHS1	SNU-407
CHS1	CL-34
CHS1	SW948
CHS1	SW1463
CHS1	NCI-H684
CHS1	LS513
CHS1	LS1034
CHS1	SW403
CHS1	LS 180
CHS1	C84
CHS1	COLO-320
CHS1	CCK-81
CHS1	KM12
CHS1	GP2d
CHS1	C80
CHS1	GP5d
CHS1	SNU-1544
CHS1	HT115
CHS1	LS411N
CHS1	C75
CHS1	JVE-127
CHS1	SNU-C4
CHS2	C10
CHS2	SNU-81
CHS2	RKO
CHS2	HCT-15
CHS2	SNU-503
CHS2	LoVo
CHS2	DLD-1
CHS2	SW48
CHS2	SNU-C2A
CHS3	TGBC18TKB
CHS3	SW837
CHS3	LS123

CHS3	HCT 116
CHS3	C2BBe1
CHS3	CL-14
CHS3	CL-11
CHS3	SW 480
CHS3	RCM-1
CHS3	NCI-H747
CHS3	OUMS-23
CHS3	SNU-1033
CHS3	CACO2
CHS3	MDST8
CHS3	JVE-253
CHS3	SW 620
CHS3	SW1417
CHS3	TT1TKB
CHS3	SNU-1197
CHS3	COLO 201
CHS3	SW1116
CHS3	SW 626
CHS3	COLO-678
CHS3	SNU-C5
CHS3	KP-363T

Table S4. The genes and coefficients of the prognostic model

gene	coefficient
NKAIN4	1.2281
NGF	1.044
HOXC6	1.0855
SLC6A1	1.0417
CLCA1	0.9492
PRG4	1.0957
PCOLCE2	1.0353

Raw imaging of WB

FDX1

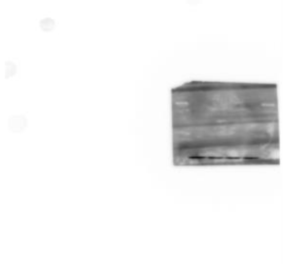

FDX1-β-actin

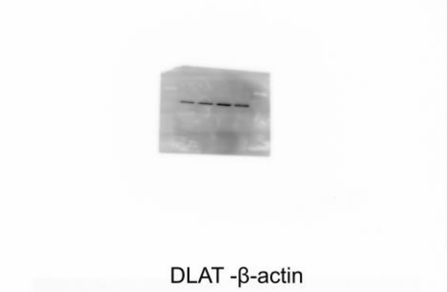

FDX1-marker

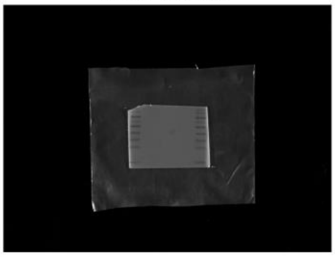

DLAT

DLAT -β-actin

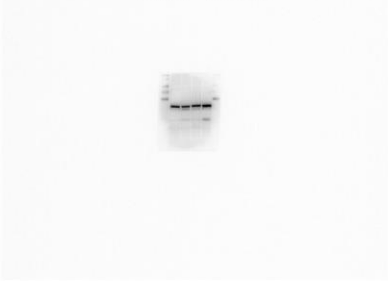

DLAT -marker

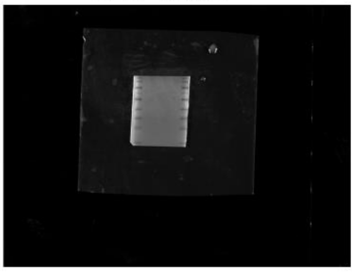

CDNK2A

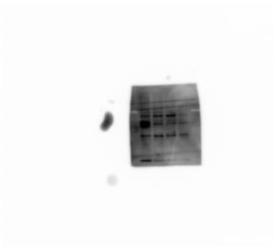

CDNK2A-β-actin

CDNK2A-marker

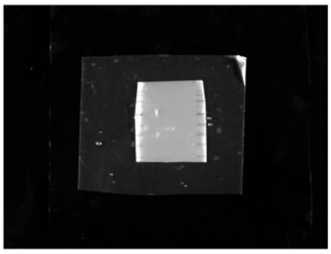

MTF1

MTF1 -β-actin

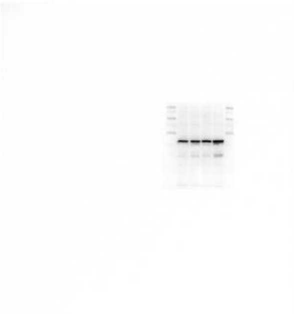

MTF1 -marker

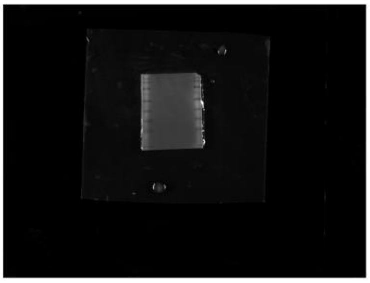

Supplement: Supplementary file 1 — Supplementary figures and tables. [file ijbsv19p3526s1.pdf]
